# Clinical biomarkers and associations with healthspan and lifespan: Evidence from observational and genetic data

**DOI:** 10.1016/j.ebiom.2021.103318

**Published:** 2021-04-01

**Authors:** Xia Li, Alexander Ploner, Yunzhang Wang, Yiqiang Zhan, Nancy L Pedersen, Patrik KE Magnusson, Juulia Jylhävä, Sara Hägg

**Affiliations:** a Department of Medical Epidemiology and Biostatistics, Karolinska Institutet, Stockholm, Sweden; b Institute of Environmental Medicine, Karolinska Institutet, Stockholm, Sweden

**Keywords:** Biomarker, Serum, Polygenic risk score, Healthspan, Lifespan

## Abstract

**Background:**

Biomarker-disease relationships are extensively investigated. However, associations between common clinical biomarkers and healthspan, the disease-free lifespan, are largely unknown. We aimed to explore the predictive values of ten biomarkers on healthspan and lifespan, and to identify putative causal mechanisms.

**Methods:**

Using data from 12,098 Swedish individuals aged 47–94 years, we examined both serum concentrations and genetically predicted levels of ten glycemic, lipid-, inflammatory, and hematological biomarkers. During a follow-up period of up to 16 years, 3681 incident cases of any chronic disease (i.e., end of healthspan) and 2674 deaths (i.e., end of lifespan) were documented. Cox regression models were applied to estimate the associations of a one standard deviation increase in biomarkers with healthspan and lifespan.

**Findings:**

Seven out of ten serum biomarkers were significantly associated with risks of any chronic disease and death; elevated glycemic biomarkers and high-density lipoprotein-related biomarkers showed the strongest detrimental (hazard ratio [HR] 1·29 [95% CI 1·24–1·34]) and protective effects (HR 0·92 [95% CI 0·89–0·96]), respectively. Genetic predisposition to elevated fasting blood glucose (FBG) was associated with increased risks of any chronic disease (HR 1·05 [95% CI 1·02–1·09]); genetically determined higher C-reactive protein correlated with lower death risks (HR 0·91 [95% CI 0·87–0·95]). Notably, the genetically proxied FBG-healthspan association was largely explained by serum FBG concentration.

**Interpretation:**

Circulating concentrations of glycemic, lipid-, and inflammatory biomarkers are predictive of healthspan and lifespan. Glucose control is a putative causal mechanism and a potential intervention target for healthspan maintenance.

**Funding:**

This study was supported by the Swedish Research Council (2015–03,255, 2018–02,077), FORTE (2013–2292), the Loo & Hans Osterman Foundation, the Foundation for Geriatric Diseases, the Magnus Bergwall Foundation, the Strategic Research Program in Epidemiology at Karolinska Institutet (SH, JJ), the China Scholarship Council, and the Swedish National Graduate School for Competitive Science on Ageing and Health. The Swedish Twin Registry is managed by Karolinska Institutet and receives funding as an infrastructure through the Swedish Research Council, 2017–00,641.

**Research in Context**

**Evidence before this study**

A literature search without language restriction was performed on December 5th, 2020, using PubMed. Searching terms were (1) “healthspan” (or “healthy lifespan” or “disease-free lifespan” or “morbidity-free lifespan” or “disease-free survival” or “morbidity-free survival”) and (2) “glucose”, “glycated hemoglobin” (or “HbA1c” or “hemoglobin A1C”), “triglyceride”, “total cholesterol” (or “cholesterol”), “high-density lipoprotein cholesterol” (“HDL”), “low-density lipoprotein cholesterol” (or “LDL”), “Apolipoprotein A1”, “Apolipoprotein B”, “C-reactive protein” (or “CRP”), or “hemoglobin”. We identified two population-based studies investigating the aforementioned biomarkers as predictors of morbidity-free survival at age 85 years. Terry et al. assessed total cholesterol and glucose among 2531 participants in the Framingham Heart Study; Newson et al. studied CRP, glucose, total cholesterol, and HDL cholesterol within 2008 individuals from the Rotterdam Study. The two studies found that participants with a lower level of total cholesterol, absence of glucose intolerance, and a lower concentration of CRP had a higher odds of survival to the age of 85 years without experiencing any major diseases, including cardiovascular diseases, stroke, cancer, and dementia. However, we found no studies providing evidence in the predictive value of the other biomarkers as well as testing the potential causal relationships between above biomarkers and healthspan by leveraging genetically predicted biomarkers.

**Added value of this study**

Using blood- and health register-based data from 12,098 Swedish individuals aged 47–94 years, we examined both serum concentrations and genetically predicted levels of ten common biomarkers in association with healthspan during follow-up periods of up to 16 years. We found that seven glycemic, lipid-, and inflammatory biomarkers were significantly associated with healthspan; elevated glycemic biomarkers and high-density lipoprotein-related biomarkers showed the strongest detrimental and protective effects, respectively. In addition, genetic predisposition to higher fasting blood glucose (FBG) was associated with increased risk of any chronic event (i.e., end of healthspan), largely explained by elevated serum FBG concentrations, suggesting glucose control as a putative causal mechanism in healthspan maintenance.

**Implications of all the available evidence**

Common clinical biomarkers that are routinely assessed at clinics to monitor disease risks could reflect the ability to maintain healthspan. These results underline the use of existing clinical biomarkers in identifying individuals with an accelerated underlying risk for aging yet without known disease diagnosis. Moreover, the putative causal mechanism of glucose control suggests that lifestyle and pharmacological interventions, such as dietary restriction, exercise, and glucose-lowering drug prescription, may help to maintain healthspan within a disease-free population

Alt-text: Unlabelled box

## 1 Introduction

Global human life expectancy has been growing over the past decades [1]. As a result, the chance of experiencing detrimental aging-related phenotypes, including dysfunction and diseases, during an individuals’ lifetime has increased. The time period that an individual spends in good health is often referred to as healthspan [2]. This concept emphasizes quality of life and is a key target for health-promoting strategies, especially among older populations [3,4]. Chronic diseases that can impair life quality irreversibly are administratively monitored through established health registers in the Nordic countries, including Sweden. One practical way to quantify healthspan from a morbidity perspective is therefore to identify the earliest onset of major chronic diseases and to report the corresponding disease-free lifetime. Recent studies investigating the genetic architecture underlying morbidity-defined healthspan has suggested healthspan as a promising phenotype for the study of human aging [5,6].

Serum biomarkers are useful instruments to predict health risks. In clinical practice, a set of serum biomarkers, including glycemic, lipid-, and inflammatory markers, are routinely assessed because of their close relationships with disease onset and progression. As disease mechanisms often correlate with the underlying aging hallmarks [[7], [8], [9]], common clinical biomarkers that are initially reflective of disease risks hold the potential to predict healthspan. Two previous studies have assessed total cholesterol, glucose tolerance, *C*-reactive protein, and high-density lipoprotein cholesterol in association with disease-free survival to the age of 85 years [10,11]. However, besides this evidence, the predictive value of serum concentrations for other biomarkers on healthspan are largely unknown.

In order to identify preventive strategies to avoid chronic diseases and maintain health in older ages, studies that identify causal mechanisms from modifiable risk factors to healthspan are needed. Genetic predispositions are not affected by environmental factors or reverse-causality. Hence, examining biomarker concentrations that are genetically determined could aid in identifying putative causal mechanisms [12,13]. Common clinical biomarkers are well-studied phenotypes with respect to their genetic susceptibility [14]; however, it is unclear how genetically predicted biomarkers could influence healthspan.

Consequently, we aim to estimate the associations of clinical serum biomarkers, including glycemic, lipid-, inflammatory, and hematological markers, with the risk of any chronic disease (i.e., end of healthspan). Secondly, we aim to examine genetically predicted biomarkers in relation to healthspan as an attempt to identify putative causal pathways. In addition, we consider a secondary outcome, death (i.e., end of lifespan), throughout the analysis because all-cause mortality is a relatively well-studied phenotype [15,16] and comparing healthspan associations against lifespan's could facilitate the comprehension of healthspan results.

## 2 Methods

### 2.1 Study population

TwinGene, a sub-cohort study within the Swedish Twin Registry (STR), recruited Swedish twins who were born between 1911 and 1958 and had participated in a prior telephone-interview survey, Screening Across the Lifespan Twin Study (SALT) in 1998–2002·[17]. In total, 12,646 individuals participated in TwinGene's baseline examination, including self-reported questionnaire query, health check-up, and blood sample collection between 2004 and 2008. In the present analyses, we excluded individuals who had unknown information on disease diagnosis, vital status, any clinical biomarker, educational attainment, body mass index (BMI), or smoking status, eventually yielding a sample size of 12,098. A total of 2560 individuals had encountered at least one chronic disease before baseline and were therefore excluded from healthspan analyses, leaving 9538 individuals (Supplementary Fig. 1).

### 2.2 Any chronic disease (end of healthspan) and death (end of lifespan)

In line with previous research [5], we defined the healthspan of each individual as the age at first occurrence of any of the following conditions (hereafter referred to as “any chronic disease”): cancer, diabetes, cardiovascular diseases (coronary heart failure [CHF], myocardial infarction [MI], stroke), chronic obstructive pulmonary disease (COPD), dementia, and death (ICD codes in Supplementary Table 1). Disease diagnosis was ascertained through linkages between STR and the Swedish National Patient Register (NPR). For each disease, we selected the earliest medical record with the corresponding diagnosis and assigned disease onset date as the admission date (inpatient record) or record date (outpatient care). All-cause mortality data, including vital status and dates of death, were obtained from the Swedish Population Register. Healthspan information was followed up through December 31st 2016; lifespan was updated through April 1, 2020.

### 2.3 Serum biomarker assessment

All TwinGene participants were instructed to fast overnight before blood collection. Blood samples were then prepared for DNA extraction and clinical chemistry tests. We investigated circulating concentrations of clinical biomarkers that reflect glycemic control (fasting blood glucose [FBG], glycated hemoglobin [HbA1c]), lipid metabolism (total cholesterol [TC], high-density lipoprotein cholesterol [HDL-C], low-density lipoprotein cholesterol [LDL-C], Apolipoprotein A1 [ApoA1], Apolipoprotein B [ApoB], triglyceride [TG]), inflammation (C-reactive protein [CRP]), and hematological function (hemoglobin [Hb]). HbA1c was assessed by ion exchange chromatography and the other clinical biomarkers were measured by a semi-automated biochemistry analyzer (Beckman Coulter, CA). Biomarkers that appeared strongly right-skewed (FBG, HbA1c, TG, and CRP) were first log-transformed; all biomarkers were then standardized (to units of 1 standard deviation [SD]).

### 2.4 Polygenic risk score (PRS) calculation

Genotypes were assessed in a total of 10,946 TwinGene participants, of whom 9835 individuals were genotyped on Illumina OmniExpress BeadChips, and 1111 individuals from complete monozygotic (MZ) twin pairs were imputed by using the genotypes of their MZ co-twins. Arrayed genetic data were then imputed against the 1000 Genomes Project phase 1 version 3 panel.

To calculate polygenic risk scores for each clinical biomarker, we referred to the genome-wide association study (GWAS) results of the UK Biobank (UKB) released by Neale's Lab (GWAS round 2) [14] and utilized the individual-level genotypes in TwinGene. For each clinical biomarker, we performed the following procedures: first, downloaded UKB GWAS summary statistics (phenotype codes and data linkage listed in Supplementary Table 2), and discarded the palindromic single-nucleotide polymorphisms (SNPs) and rare variants (minor allele frequency [MAF] < 5%); second, identified post-imputation SNPs that passed the quality control criteria in TwinGene: 1) non-palindromic SNPs, 2) imputation quality R square >0·8, and 3) MAF>=5%; third, filtered UKB summary statistics by keeping quality controlled SNPs, and performed linkage disequilibrium (LD)-based clumping to select independent genetic variants using PLINK1·9, where reference panel, significance threshold for index SNPs, secondary significance threshold, LD r^2^ threshold, and physical distance threshold were set to 1000 Genomes EUR, 1, 1, 0·1, and 1000 kb, respectively; fourth, selected the index SNPs with a GWAS significant P value (<5E-8), calculated the PRSs as the weighted sum of biomarker-elevating alleles for each TwinGene participant, and lastly standardized PRS values to SD units.

In summary, PRSs were derived from a different number of SNPs (from 63 for FBG to 590 for HDL-C), were positively associated with the corresponding serum biomarker (*P* values from 1·49e-44 to 1·16e-261), and explained varying degrees of phenotypic variance (1·9% - 8·8%; Supplementary Table 3).

### 2.5 Covariate assessment

BMI was derived from TwinGene's baseline physical measurements and calculated as weight (kg) divided by height (m) squared. Statin usage was self-reported in TwinGene's baseline survey. The number of attained years of education and smoking status (never smokers vs. ever smokers) were ascertained through self-reported information from the SALT study, which was conducted about six years prior to TwinGene.

### 2.6 Statistical methods

First, we described the baseline characters using mean (SD), median (interquartile range [IQR]), and frequency (proportion) whenever appropriate. Correlations between clinical biomarker pairs were quantified by Pearson correlation coefficients. Smooth hazard functions (i.e. instantaneous incidence rates) for outcomes of interest were estimated non-parametrically using the *R* package bshazard [18].

Next, we applied proportional hazard Cox regression models to estimate the hazard ratios (HRs), which indicate the associations between serum biomarkers at baseline and the hazard of outcomes (any chronic disease and death) during follow-up among all participants, men, and women, respectively. To account for relatedness within twin pairs, we used robust standard errors in the Cox models. Each participant was followed from the baseline age until the age of any chronic disease, death, or the end of follow-up. All models were adjusted for age (by using attained age as underlying time scale), sex, birth year category in decades, educational attainment, BMI, smoking status, and statin usage. To control the false discovery due to multiple testing, we corrected P values using Benjamini-Hochberg false discovery rate (FDR). In addition, we analyzed multiple biomarkers in the same model simultaneously and conducted variable selection using a stepwise procedure. We started with a Cox model where all serum biomarkers were included. Then we used Akaike information criterion (AIC) to select a model through both a forward and backward strategy.

To estimate genetic associations between biomarker PRSs and outcomes of interest, we adopted a similar Cox regression approach, with a few modifications: survival models were adjusted for age (through treating attained age as time scale), sex, birth year category, and the first ten genomic PCs. Two additional models were estimated to explore potential pathways that underlie the association between FBG PRS and healthspan, with further adjustment for (1) BMI and serum TG, LDL-C, HDL-C, CRP, Hb, and (2) serum FBG. Similar models were applied to CRP PRS and lifespan, with further adjustment for (1) BMI and serum TG, LDL-C, HDL-C, FBG, Hb, and (2) serum CRP.

To further demonstrate the validity of findings related to healthspan and to increase the generalizability, we replicated the statistically significant associations in an independent cohort, the Swedish Adoption/Twin Study of Aging (SATSA) [19]. Study characteristics in SATSA and analytical procedures are detailed in Supplementary Method 1.

Lastly, we performed two sensitivity analyses to test the robustness of our findings. First, to test whether the associations between biomarkers and healthspan were solely driven by one individual disease, we performed a “leave-one-disease-out” sensitivity analysis. We changed the definition of healthspan seven times by excluding one disease out of the healthspan-ending event list at a time. Next, to rule out the effects due to outliers, we excluded participants with serum biomarkers values exceeding the range of mean±3SD.

Statistical significance was defined as a *P* value or a FDR-corrected P value of less than 0·05. All analyses were implemented in *R* 3·6·1 and PLINK1·9.

### 2.7 Ethics statement

Informed consent was obtained from all participants. The study was approved by the Swedish Ethical Review Authority in Stockholm (Dnr 2016/1888–31/1).

### 2.8 Role of the funding source

The funders of the study had no role in study design, data collection, data analysis, data interpretation, or manuscript drafting. All authors had full access to all the data in the study and accept responsibility to submit for publication.

## 3 Results

### 3.1 Baseline characteristics of study participants

Among 12,098 participants, 9300 (76·9%) constituted complete twin pairs, 5469 (45·2%) were men, average age was 64·9 years, and 9538 (78·8%) were free from any chronic disease at baseline (Table 1). Men presented higher proportions of prevalent diabetes and cardiovascular diseases (including diabetes, MI, CHF, and stroke) at baseline than women (*P*<0·001) (Supplementary Table 4).

**Table 1:** Characteristics of study participants.

	Participants in healthspan analysis	Participants in lifespan analysis
Number of individuals	9538	12,098
Number of twin pairs/individuals 1		
MZ pairs	880 (18·5%)	1307 (21·6%)
Same-sex DZ pairs	1159 (24·3%)	1768 (29·2%)
Opposite-sex DZ pairs	1005 (21·1%)	1573 (26·0%)
Single individuals 2	3446 (36·1%)	2798 (23·1%)
**Baseline characteristics**^3^**(N [%] or Mean [SD])**		
Men	4110 (43·1%)	5469 (45·2%)
Age (year)	63·9 (7·8)	64·9 (8·1)
Educational attainment (year)	10·9 (3·2)	10·8 (3·2)
BMI (kg/m^2^)	25·7 (3·8)	25·9 (3·9)
Ever-smokers	5233 (54·9%)	6776 (56·0%)
Statin-users	594 (6·2%)	1158 (9·6%)
**Prevalent diseases at baseline (N [%])**		
Cancer	0 (0·0%)	1221 (10·1%)
Diabetes	0 (0·0%)	482 (4·0%)
MI	0 (0·0%)	686 (5·7%)
CHF	0 (0·0%)	219 (1·8%)
Stroke	0 (0·0%)	348 (2·9%)
COPD	0 (0·0%)	192 (1·6%)
Dementia	0 (0·0%)	21 (0·2%)
Any prevalent chronic disease 4	0 (0·0%)	2560 (21·2%)
**Serum biomarkers at baseline (Mean [SD] or Median [IQR])**		
FBG (mmol/L; median)	5·3 (0·7)	5·3 (0·8)
HbA1c (%; median)	4·6 (0·4)	4·7 (0·4)
TG (mmol/L; median)	1·1 (0·8)	1·2 (0·8)
TC (mmol/L)	5·9 (1·1)	5·8 (1·1)
HDL-C (mmol/L)	1·4 (0·4)	1·4 (0·4)
LDL-C (mmol/L)	3·9 (0·9)	3·8 (1·0)
ApoA1 (g/L)	1·7 (0·3)	1·6 (0·3)
ApoB (g/L)	1·09 (0·24)	1·08 (0·24)
CRP (mg/L; median)	1·6 (2·5)	1·7 (2·7)
Hb (g/L)	142·5 (11·5)	142·3 (11·9)
**Follow-up information (N [%] or Median [IQR])**		
Follow-up time (year)	9·5 (3·4)	13·0 (1·5)
Number of incident cases	3681 (38·6%)	2674 (22·1%)
Onset age of incident cases / age at death	72·3 (11·8)	81·7 (12·1)

1 Two twin pairs with unknown zygosity were not shown in the table.

2 Individuals whose co-twins were not included.

3 Baseline survey refers to the blood sampling conducted in 2004–2008.

4 Including cancer, diabetes, MI, CHF, stroke, COPD, and dementia. MZ monozygotic, DZ dizygotic, N number of individuals, SD standard deviation, IQR interquartile range, MI myocardial infarction, CHF coronary heart failure, COPD chronic obstructive pulmonary disease, FBG fasting blood glucose, HbA1c hemoglobin A1C, TC total cholesterol, HDL-C high-density lipoprotein cholesterol, LDL-C low-density lipoprotein cholesterol, ApoA1 Apolipoprotein A1, ApoB Apolipoprotein B, TG triglyceride, CRP C-reactive protein, Hb hemoglobin.

Pearson correlations analysis showed glycemic and lipid biomarkers constituted three correlation clusters (coefficients higher than 0·6), namely 1) FBG and HbA1c, 2) TC, LDL-C, and ApoB, and 3) HDL-C and ApoA1 (Supplementary Fig. 2).

### 3.2 Any chronic disease (end of healthspan) and death (end of lifespan)

**Fig. 1:**
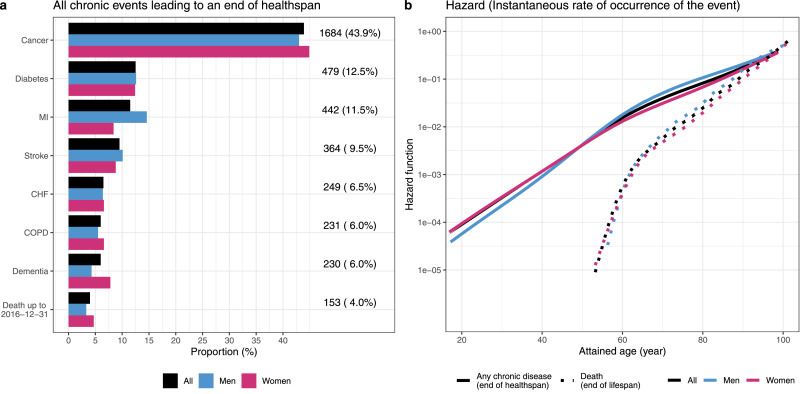
Events leading to an end of healthspan and smooth hazard functions of any chronic event and death. Panel a illustrated the event-specific number of cases that lead to an end of healthspan. Panel *b* presented the hazard of experiencing any chronic event and death across age spectrum. Results derived from all, men, and women were colored in black, blue, and red (For interpretation of the references to color in this figure legend, the reader is referred to the web version of this article.).

During a median follow-up time of 9·5 years, 3681 (38·6%) individuals experienced at least one incident event (Table 1). The most frequent events leading to an end of healthspan were cancer, diabetes, and MI (Fig. 1a). A total of 2674 (22·1%) deaths were documented during a median follow-up time of 13·0 years (Table 1). Median ages of any chronic disease and of death were 72·3 and 81·7, respectively (Supplementary Table 5).

We found the risks of encountering any chronic disease and death increased exponentially with age, with the rate of increase slowing at the age of 60. The hazard of any chronic disease was higher than the hazard of death throughout, but increased at a slower speed, with almost-convergence at the very end of the observed age range (Fig. 1b).

### 3.3 Serum biomarkers, healthspan and lifespan

**Fig. 2:**
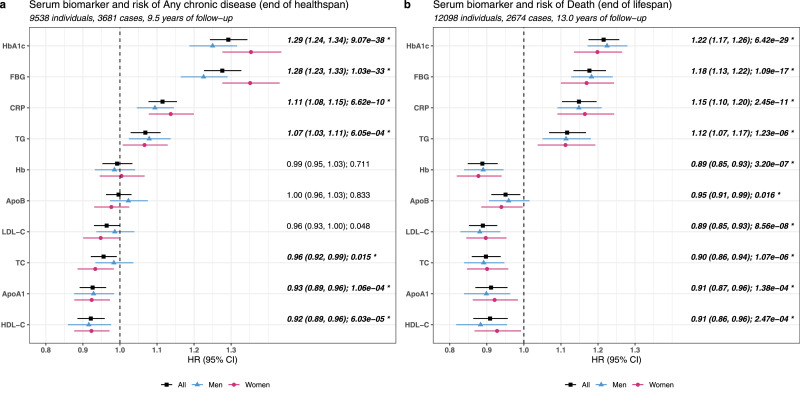
Associations of serum clinical biomarkers with healthspan and lifespan. Panel a–b illustrated the associations, quantified as HR (95% CI), of serum biomarkers with any chronic event and death, respectively. HRs indicate the relative risk of experiencing any chronic event or death associated with one-SD increase in serum biomarker concentrations. In each panel, we used dots and lines to present HR point estimates and confidence intervals; estimations derived from all, men, and women were colored in black, blue, and red. In panel a, we sorted the biomarkers along y scale by the magnitudes of HRs observed among all participants, from the highest (risk factor) to the lowest (protective factor); panel b adopted the same biomarker order as in panel a. *P* values that were significant after FDR-correction were marked by * (For interpretation of the references to color in this figure legend, the reader is referred to the web version of this article.).

We observed that higher circulating levels of HbA1c, FBG, CRP, and TG were indicative of a higher risk of healthspan ending (Fig. 2a and Supplementary Table 6); in contrast, increased levels of HDL-C, ApoA1, TC were associated with a lower risk of any chronic disease; no statistically significant evidence of association was observed after multiple testing correction for LDL-C, Hb and ApoB. Of all healthspan-detrimental biomarkers, glycemic biomarkers exhibited the largest effect sizes, with a one-SD increase in HbA1c associated with a 29% increased risk (HR 1·29, 95% CI 1·24–1·34]). Within all healthspan-beneficial biomarkers, those related to HDL metabolism showed the greatest effects, in which a one-SD increase in HDL-C was associated with a 8% decreased risk (HR 0·92, 95% CI 0·89–0·96). In an independent Swedish cohort, SATSA, we also observed statistically significant results for serum FBG, CRP, and HDL-C in association with heathspan, as well as directionally consistent evidence for serum HbA1c, TC, and ApoA1 (Supplementary Table 7).

With regard to lifespan associations, we found all clinical biomarkers were statistically significantly associated with death (Fig. 2b and Supplementary Table 6); specifically, Hb, ApoB, and LDL-C were inversely associated with death risks, while the other biomarkers showed similar patterns as with healthspan.

In the multiple-biomarker models, the two sets of biomarkers selected were only slightly different. Specifically, FBG, HbA1c, TG, TC, and CRP provided independent information associated with both healthspan and lifespan (Supplementary Table 8).

### 3.4 Biomarker PRSs, healthspan and lifespan

**Fig. 3:**
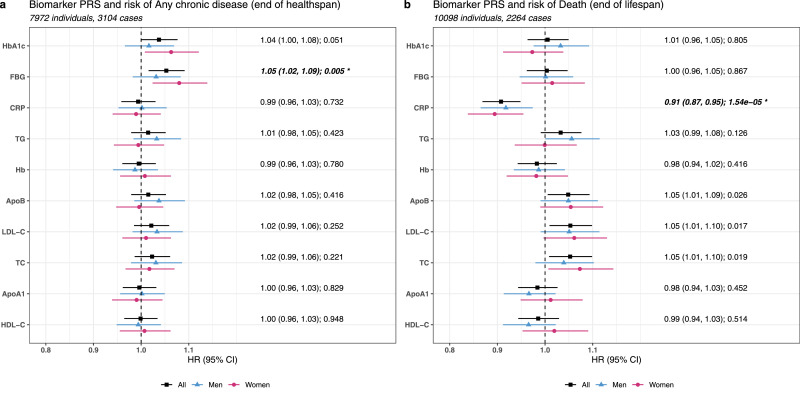
Associations of biomarker PRSs with healthspan and lifespan. Panel a–b illustrated the associations, quantified as HR (95% CI), of biomarker PRSs with any chronic event and death, respectively. HRs indicate the relative risk of experiencing any chronic event or death associated with one-SD increase in biomarker PRSs (i.e., genetic predisposition to elevated biomarker levels). In each panel, we used dots and lines to present HR point estimates and confidence intervals; estimations derived from all, men, and women were colored in black, blue, and red. Panel a–b adopted the same biomarker order as in Fig. 2 panel a. *P* values that were significant after FDR-correction were marked by *.

We observed a statistically significant relationship between FBG PRS and the risk of any chronic disease with an HR of 1·05 (1·02, 1·09), meaning a one-SD increase in genetic predisposition to elevated blood glucose level was associated with a 5% higher risk (HR 1·05, 95% CI 1·02–1·09). The other glycemic PRS, HbA1c PRS, showed a weak association in the same direction, but was not statistically significant. Results for other clinical biomarkers PRSs showed either null or very weak associations (Fig. 3a and Supplementary Table 9).

Additional models showed that the effect of FBG PRS on any chronic disease was independent of other non-glycemic markers, including BMI, as well as serum TG, HDL-C, LDL-C, CRP, and Hb (HR 1·05, 95% CI 1·01–1·09). However, controlling for serum FBG led to a substantial attenuation in effect estimate (HR 1·01, 95% CI 0·98–1·05; Table 2). The result suggests FBG PRS-healthspan association could be explained by serum FBG level to a large extent.

**Table 2:** Associations of FBG PRS and CRP PRS with additional adjustment.

	Original model		Additional Model 1^1^		Additional Model 2 2	
	HR (95%CI)	P	HR (95%CI)	P	HR (95%CI)	P
FBG PRS and any chronic disease	1·05 (1·02, 1·09)	0·005	1·05 (1·01, 1·09)	0·008	1·01 (0·98, 1·05)	0·526
CRP PRS and death	0·91 (0·87, 0·95)	1·5e-5	0·90 (0·86, 0·94)	2·5e-6	0·86 (0·82, 0·90)	3·7e-10

1 FBG PRS: Original model + adjustment for BMI and serum TG, LDL-C, HDL-C, CRP, Hb; CRP PRS: Original model + adjustment for BMI and serum TG, LDL-C, HDL-C, FBG, Hb.

2 FBG PRS: Original model + adjustment for serum FBG; CRP PRS: Original model + adjustment for serum CRP.

Lifespan associations showed statistically significant evidence suggesting genetically predicted higher CRP was associated with a lower risk of death, with an HR of 0·91 (95% CI 0·87–0·95) estimated for a one-SD increase in CRP PRS. HR estimates also suggested an increase in the PRS predisposing to higher circulating levels of TC, LDL-C, and ApoB increased the risk of death, albeit with statistically non-significant P values after multiple testing correction. No robust evidence of lifespan association was observed for the other PRSs (Fig. 3b and Supplementary Table 9).

Further, we found neither serum CRP nor other markers (BMI and serum TG, LDL-C, HDL-C, FBG, and Hb) could explain the beneficial survival effect of CRP PRS (Table 2). Particularly, the results remained intact when we replaced the whole-genome CRP PRS derived from 95 SNPs with a regional CRP PRS derived from 4 SNPs located in the CRP gene region (Supplementary Table 10).

### 3.5 Sensitivity analyses

Generally, the directions of biomarker associations were consistent in the “leave-one-disease-out” sensitivity analysis; most associations were more pronounced (HR further from 1) after excluding cancer cases, while less pronounced (HR closer to 1) after excluding diabetes cases. Particularly, the effects of glycemic biomarkers were largely attenuated with the exclusion of diabetes. The exclusion of diseases other than cancer and diabetes barely changed the results (Supplementary Fig. 3 and Supplementary Table 11). After exclusion of outliers, we observed slightly changed point estimates of HRs and CIs (Supplementary Fig. 4).

## 4 Discussion

Using a wealth of genetic and phenotypic data from the Swedish population-based cohort TwinGene we examined ten common clinical biomarkers in association with two aging phenotypes, healthspan and lifespan. We found that seven out of ten serum biomarkers predicted future risks of both any chronic disease and death, with elevated glycemic biomarkers and HDL-related biomarkers showing the strongest detrimental and protective effects, respectively. Moreover, genetically predicted FBG was positively associated with healthspan, and the association was largely explained by serum FBG concentrations, suggesting glucose control is a putative causal mechanism in healthspan maintenance.

Unlike lifespan, which has a universal and unambiguous definition, the healthspan concept comes with no consensus [2,20,21]. As “healthy” is a subjective concept, and healthy-unhealthy states could sometimes transform mutually, the measurement of “healthspan” is often challenging [21]. Here we started from the morbidity perspective to quantify healthspan mainly because of three reasons. First, chronic diseases often indicate impaired organismal health status, both physiologically and functionally, and are unlikely to be fully cured. Second, the resource of nationwide health registers in Sweden, documenting inpatient and out-patient records, made the establishment of morbidity-defined healthspan possible. Third, a morbidity-assessed healthspan phenotype has been studied previously by Zenin et al. in a large-scale population cohort, UKB [5]. Similar to UKB, cancer is the most common reason accounting for healthspan ending, followed by diabetes and MI. Two previous studies have examined a small number of biomarkers (TC, HDL-C, glucose tolerance, CRP) in relation to disease-free survival at the age of 85 years [10,11]. Both studies took cardiovascular diseases (CVDs), cancer, and dementia into account when defining disease-free survival, while we further incorporated diabetes and COPD in healthspan assessment. We noticed that diabetes diagnosis might play an important role in determining healthspan, as excluding diabetes from the list of healthspan-ending events attenuated the associations of both diabetes (FBG and HbA1c) and CVD (lipids) markers in the sensitivity analyses. Prediabetes being a CVD risk factor could partially explain our observation [22]. Overall, this suggested chronic diseases have overlapping mechanisms and reinforce the need to examine risk factors of aging.

Among all included blood assays, glycemic biomarkers are among the strongest risk factors of both any chronic disease and death. Hyperglycemia is often accompanied by insulin resistance, and in observational studies has been associated with increased risks of several chronic conditions, including liver, pancreatic, and breast cancer, CVDs, and neurodegenerative diseases [[23], [24], [25], [26], [27]]. Genetic predisposition represents lifetime exposure and is not affected by lifestyle factors, hence an ideal tool to identify underlying causal mechanisms [12,13]. Previous studies found genetic predisposition to elevated blood glucose was weakly associated with higher risks of breast cancer, arterial stiffness, and Alzheimer's disease [[28], [29], [30]], yet there was no evidence concerning the association between genetically determined FBG and healthspan prior to our study. We found the elevated FBG PRS was a risk factor of any chronic disease and, noticeably, serum FBG levels could largely explain the FBG PRS - healthspan relationship. Taken together, these results suggest a putative causal role of glucose control and that lifestyle and pharmacological interventions, such as dietary restriction, exercise, and glucose-lowering drug prescription, may help to maintain healthspan within a disease-free population where 90% of participants’ FBG levels ranged from 4.5 (corresponds to “normal” [31]) to 6.8 (corresponds to “prediabetes” [31]) mmol/L.

In observational studies, increased circulating concentrations of LDL and HDL are recognized as protective and risk factors of CVDs, respectively [32,33]. However, only the LDL-CVD relationship has been confirmed to be causal in Mendelian randomization (MR) studies and randomized controlled trials (RCTs), [34,35] while null causal associations were found for HDL [36,37]. Our study focused on a series of chronic diseases rather than CVD alone, and found serum concentration of HDL and their genetic predispositions were associated with healthspan/lifespan in a similar directional manner as with CVD evidence. An inconsistent relationship was observed for serum LDL, where we found a negative association between LDL and healthspan/lifespan, as opposed to the positive LDL-CVD association. Similar to our finding, the negative LDL-mortality correlation has been shown in several observational cohorts [16,38,39], where low levels of LDL are indicative of a higher risk of death, especially among older populations and non-statin users. We also found that our participants with low serum LDL tended to present with other mortality risk factors such as high glucose level and smoking. Uncontrolled confounders could partly explain the negative observational association especially since PRS analysis showed an opposite result. Nevertheless, the direction of our results, albeit non-significant after multiple testing correction, still supported a positive causal relationship between LDL and death, while our study, along with others [16,38,39], introduce the need to re-evaluate the direction of mortality predictive value for LDL among the general aging population.

Elevated circulating CRP is a risk factor for CVDs; [40] yet genetically predicted CRP was not associated with coronary heart disease [41,42]. Interestingly, our results showed that an elevated serum CRP concentration was associated with higher risks of any chronic disease and death, whereas genetic predisposition to higher CRP indicated lower death risk. In line with our findings, another large-scale population study also found contradicting directions when it comes to mortality associations using serum and genetically proxied CRP, respectively [16]. We found the effect of PRS CRP on death cannot be explained by serum CRP level. Since CRP is a protein produced in response to inflammatory stimuli [43], one possible explanation is that elevated levels of serum CRP and genetically determined CRP reflect different health conditions. An increased concentration of serum CRP often indicates underlying acute infection or chronic endogenous inflammation; while genetic predisposition to CRP could influence the response to upstream pro- and anti-inflammatory pathways important for chronic disease control [44]. Further investigations to test this hypothesis are certainly warranted.

The present analysis comes with a few strengths. We used the data from health registers in Sweden that have nationwide coverage, making the assessment of morbidity-based healthspan information during long-term follow-up possible. Next, we used external GWAS summary statistics in PRSs calculation, avoiding overfitting problems to a large extend. Meanwhile, we acknowledge several limitations. The measurement of healthspan only took eight chronic conditions into account. Our results may not be directly generalized to another healthspan outcome that incorporates other diseases. In line with previous studies [5,6], we found that diabetes plays an important role in the healthspan phenotype, based on both demographic and genetic evidence. We acknowledge that the strong association between glycemic biomarkers and healthspan in our material is due to the inclusion of diabetes as terminating event in the definition of healthspan adopted, which is highlighted by the results of our sensitivity analysis. It is therefore possible that a different healthspan definition may result in different findings. Regardless, our evidence supports diabetes as an important terminating event for overall healthspan in a generally disease-free population, and FBG as an actionable target to improve healthspan. Second, the generalizability of the present analysis is limited since we only studied a middle-aged Swedish population, which was in relatively good health at baseline.

In conclusion, we demonstrated that circulating glycemic, lipid-, and inflammatory biomarkers are associated with healthspan and lifespan. Particularly, glucose control is a putative causal mechanism and a potential intervention target for healthspan maintenance.

## Declaration of Competing Interest

All authors declare no competing interest.

## Appendix Supplementary materials

Image, application 1

Image, application 2
