# Supplementary material for: Clinical biomarkers and associations with healthspan and lifespan: Evidence from observational and genetic data
Source: eBioMedicine. 2021 Apr 1;66:103318. doi: 10.1016/j.ebiom.2021.103318 (PMC8047464; doi:10.1016/j.ebiom.2021.103318)
Supplement: Supplementary file 1 [file mmc1.pdf]

Supplementary files

Contents

Contents 1

Supplementary Method 1 Validation in SATSA 2

Supplementary Table 1 ICD codes related to the chronic diseases 3

Supplementary Table 2 Phenotype codes and data linkage of UKB summary statistics 5

Supplementary Table 3 Serum biomarkers and PRSs 6

Supplementary Table 4 Sex-specific characteristics of study participants 7

Supplementary Table 5 Number of prevalent and incident events in all participants, men, and women 9

Supplementary Table 6 Associations of serum clinical biomarkers with healthspan and lifespan in all participants, men, and women 11

Supplementary Table 7 Validation of biomarker-healthspan associations in SATSA 14

Supplementary Table 8 Multiple-biomarkers regression 15

Supplementary Table 9 Associations of biomarker PRSs with healthspan and lifespan in all participants, men, and women 16

Supplementary Table 10 Associations of regional CRP PRS and death 18

Supplementary Table 11 Leave-one-disease-out sensitivity analysis 19

Supplementary Figure 1 Study design and study population 26

Supplementary Figure 2 Correlations of serum biomarkers 27

Supplementary Figure 3 Leave-one-disease-out sensitivity analysis 28

Supplementary Figure 4 Sensitivity analysis with the exclusion of serum biomarker outliers 29

Reference 30

Supplementary Method 1 Validation in SATSA

SATSA is a sub-cohort of the Swedish Twin Registry (STR) [1], but consists of completely independent participants compared to those participating in TwinGene. A maximum of 844 SATSA participants (341 men and 503 women) had the following information: 1) at least one serum biomarker assessed, 2) healthspan derived from disease diagnoses, 3) follow-up on all-cause mortality (as death is one of the healthspan-terminating conditions). In addition, a total of 542 SATSA participants had their genotypes assessed.
Briefly, we replicated the following procedures in SATSA: 1) developing the healthspan phenotype using the same definition in TwinGene; 2) selecting the first available biomarker assessment when repeated measurements are available (average age of 63.5 years), log transforming where required (FBG, HbA1c, TG, and CRP), and rescaling all biomarker values to units of one standard deviation; 3) using the same genetic loci and GWAS weights in TwinGene analysis to calculate the polygenic risk score (PRS) for FBG among SATSA participants; 4) re-examining the statistically significant biomarker-healthspan associations identified in TwinGene using the same statistical methods.

Supplementary Table 1 ICD codes related to the chronic diseases

	ICD-7 -1967	ICD-8 1968-1986	ICD-9 1987-1996	ICD-10 1997-
Diabetes	260 Diabetes mellitus	250 Diabetes mellitus	250 Diabetes mellitus	E10 Insulin-dependent diabetes mellitus<br>E11 Non-insulin-dependent diabetes mellitus<br>E12 Malnutrition-related diabetes mellitus<br>E13 Other specified diabetes mellitus<br>E14 Unspecified diabetes mellitus
CHF	434·1 Congestive heart failure<br>434·2 Left ventricular failure	427·0 Congestive heart failure<br>427·1 Left ventricular failure	428 Heart failure	I50 Heart failure
Cancer	140-207	140-209	140-208	C00-C97<br>B21
MI	420 Arteriosclerotic heart disease, including coronary disease	410 Acute myocardial infarction<br>411 Other acute and subacute forms of ischaemic heart disease<br>412 Chronic ischaemic heart disease<br>414 Asymptomatic ischaemic heart disease	410 Acute myocardial infarction<br>411 Other acute and subacute forms of ischaemic heart disease<br>412 Old myocardial infarction<br>414 Other forms of chronic ischaemic heart disease	I21 Acute myocardial infarction<br>I22 Subsequent myocardial infarction<br>I23 Certain current complications following acute myocardial infarction<br>I24 Other acute ischaemic heart diseases<br>I25 Chronic ischaemic heart disease
Stroke	330 Subarachnoid haemorrhage<br>331 Cerebral haemorrhage<br>332 Cerebral embolism and thrombosis<br>334 Other and ill-defined vascular lesions affecting central nervous system	430 Subarachnoid haemorrhage<br>431 Cerebral haemorrhage<br>433 Cerebral thrombosis<br>434 Cerebral embolism<br>436 Acute but ill-defined cerebrovascular disease	430 Subarachnoid haemorrhage<br>431 Intracerebral haemorrhage<br>432 Other and unspecified intracranial haemorrhage<br>434 Occlusion of cerebral arteries<br>436 Acute but ill-defined cerebrovascular disease	I60 Subarachnoid haemorrhage<br>I61 Intracerebral haemorrhage<br>I62 Other nontraumatic intracranial haemorrhage<br>I63 Cerebral infarction<br>I64 Stroke, not specified as haemorrhage or infarction
COPD	501 Bronchitis unqualified<br>502 Chronic bronchitis	490 Bronchitis, unspecified<br>491 Chronic bronchitis<br>492 Emphysema	490 Bronchitis, not specified as acute or chronic<br>491 Chronic bronchitis<br>492 Emphysema<br>496 Chronic airways obstruction, not elsewhere classified	J40 Bronchitis, not specified as acute or chronic<br>J41 Simple and mucopurulent chronic bronchitis<br>J42 Unspecified chronic bronchitis<br>J43 Emphysema<br>J44 Other chronic obstructive pulmonary disease
Dementia	304 Senile psychosis<br>305 Presenile psychosis<br>306 Psychosis with cerebral arteriosclerosis	290 Senile and presenile dementia<br>293·0 Cerebral arteriosclerosis<br>293·1 Other cerebrovascular disturbances	290·0 Senile dementia, uncomplicated<br>290·1 Presenile dementia<br>290·4 Arteriosclerotic dementia<br>290·8 Other specified senile psychotic conditions	F00 Dementia in Alzheimer's disease<br>F01 Vascular dementia<br>F02 Dementia in other diseases classified elsewhere<br>F03 Unspecified dementia

	290·9 Unspecified senile psychotic condition	F05·1 Delirium superimposed on dementia
	294·1 Dementia in conditions classified elsewhere	G30 Alzheimer's disease
	331·0 Alzheimer's disease	G31·1 Senile degeneration of brain, not elsewhere classified
	331·1 Pick's disease	G31·8A Lewy body dementia
	331·2 Senile degeneration of brain	
	331·9 Cerebral degeneration, unspecified	

MI myocardial infarction, CHF coronary heart failure, COPD chronic obstructive pulmonary disease.

Supplementary Table 2 Phenotype codes and data linkage of UKB summary statistics

	Phenotype Code	Phenotype Description	Dropbox File
FBG GWAS	30740_raw	Glucose (mmol/L)	https://www.dropbox.com/s/2ay8s1ctsm0m3qv/30740_raw.gwas.imputed_v3·both_sexes.tsv.bgz?dl=0
HbA1c GWAS	30750_raw	Glycated haemoglobin (mmol/mol)	https://www.dropbox.com/s/c4sgeauksc2cizh/30750_raw.gwas.imputed_v3·both_sexes.tsv.bgz?dl=0
TG GWAS	30870_raw	Triglycerides (mmol/L)	https://www.dropbox.com/s/0tdxu9g7itbct6m/30870_raw.gwas.imputed_v3·both_sexes.tsv.bgz?dl=0
TC GWAS	30690_raw	Cholesterol (mmol/L)	https://www.dropbox.com/s/a634mjw9gpitjy4/30690_raw.gwas.imputed_v3·both_sexes.tsv.bgz?dl=0
LDL-C GWAS	30780_raw	LDL direct (mmol/L)	https://www.dropbox.com/s/2msvdv4axfz362b/30780_raw.gwas.imputed_v3·both_sexes.tsv.bgz?dl=0
ApoB GWAS	30640_raw	Apoliprotein B (g/L)	https://www.dropbox.com/s/otgrue1c6ir0r87/30640_raw.gwas.imputed_v3·both_sexes.tsv.bgz?dl=0
HDL-C GWAS	30760_raw	HDL cholesterol (mmol/L)	https://www.dropbox.com/s/sn30890f64p0htu/30760_raw.gwas.imputed_v3·both_sexes.tsv.bgz?dl=0
ApoA GWAS	30630_raw	Apoliprotein A (g/L)	https://www.dropbox.com/s/jkedtnnxmx2ccfy/30630_raw.gwas.imputed_v3·both_sexes.tsv.bgz?dl=0
CRP GWAS	30710_raw	C-reactive protein (mg/L)	https://www.dropbox.com/s/i8hg3jlc8fetcob/30710_raw.gwas.imputed_v3·both_sexes.tsv.bgz?dl=0
Hb GWAS	30020_raw	Haemoglobin concentration	https://www.dropbox.com/s/rvpbvobej0f3ryl/30020_raw.gwas.imputed_v3·both_sexes.tsv.bgz?dl=0

Additional information can be found at https://docs.google.com/spreadsheets/d/1kvPoupSzsSFBNSztMzl04xMoSC3Kcx3CrjVf4yBmESU/edit?ts=5b5f17db#gid=178908679

Supplementary Table 3 Serum biomarkers and PRSs

Phenotype (unit: SD)	PRS (unit: SD)	Number of SNPs included in the PRS calculation	Phenotype-PRS association (beta coefficient)	Phenotype-PRS association (P value)	phenotypic variance explained by PRS
Serum FBG	FBG PRS	63	0·11	1·49e-44	1·9%
Serum HbA1c	HbA1c PRS	310	0·18	3·12e-115	4·9%
Serum TG	TG PRS	425	0·25	4·93e-169	7·2%
Serum TC	TC PRS	391	0·24	1·14e-136	5·7%
Serum LDL-C	LDL-C PRS	355	0·25	1·57e-138	5·9%
Serum ApoB	ApoB PRS	410	0·27	9·53e-176	7·5%
Serum HDL-C	HDL-C PRS	590	0·30	1·16e-241	8·8%
Serum ApoA1	ApoA1 PRS	514	0·28	4·18e-207	7·7%
Serum CRP	CRP PRS	95	0·14	1·00e-134	5·7%
Serum Hb	Hb PRS	503	0·18	2·92e-94	3·1%

FBG fasting blood glucose, HbA1c hemoglobin A1C, TC total cholesterol, HDL-C high-density lipoprotein cholesterol, LDL-C low-density lipoprotein cholesterol, ApoA1 Apolipoprotein A1, ApoB Apolipoprotein B, TG triglyceride, CRP C-reactive protein, Hb hemoglobin.

Supplementary Table 4 Sex-specific characteristics of study participants

	Healthspan cohort			Lifespan cohort		
	Men	Women	P_{sex}	Men	Women	P_{sex}
Number of individuals	4110	5428		5469	6629	
Number of twin pairs/individuals [1]						
MZ pairs	340 (16·5%)	540 (19·9%)		538 (19·7%)	769 (23·2%)	
Same-sex DZ pairs	421 (20·5%)	738 (27·2%)		720 (26·3%)	1048 (31·6%)	
Single individuals [2]	2584 (62·9%)	2872 (52·9%)		2949 (53·9%)	2995 (45·2%)	
**Baseline characteristics [3] (N [%] or Mean [SD])**						
Age (year)	64·0 (7·6)	63·8 (7·9)	0·218	65·3 (8·0)	64·6 (8·2)	<0·001
Education (year)	10·9 (3·2)	10·9 (3·2)	0·346	10·7 (3·3)	10·8 (3·2)	0·027
BMI (kg/m^2)	26·0 (3·4)	25·5 (4·1)	<0·001	26·2 (3·4)	25·6 (4·2)	<0·001
Ever-smokers	2433 (59·2%)	2800 (51·6%)	<0·001	3311 (60·5%)	3465 (52·3%)	<0·001
Statin-users	266 (6·5%)	328 (6·0%)	0·393	626 (11·4%)	532 (8·0%)	<0·001
**Prevalent diseases at baseline (N [%])**						
Cancer	0 (0·0%)	0 (0·0%)		536 (9·8%)	685 (10·3%)	0·347
Diabetes	0 (0·0%)	0 (0·0%)		293 (5·4%)	189 (2·9%)	<0·001
MI	0 (0·0%)	0 (0·0%)		502 (9·2%)	184 (2·8%)	<0·001
CHF	0 (0·0%)	0 (0·0%)		133 (2·4%)	86 (1·3%)	<0·001
Stroke	0 (0·0%)	0 (0·0%)		192 (3·5%)	156 (2·4%)	<0·001
COPD	0 (0·0%)	0 (0·0%)		86 (1·6%)	106 (1·6%)	0·942
Dementia	0 (0·0%)	0 (0·0%)		15 (0·3%)	6 (0·1%)	
Any prevalent chronic disease [4]	0 (0·0%)	0 (0·0%)		1359 (24·8%)	1201 (18·1%)	<0·001
**Serum biomarkers at baseline (Mean [SD] or Median [IQR])**						
FBG (mmol/L; median)	5·4 (0·7)	5·2 (0·7)	<0·001	5·5 (0·9)	5·2 (0·7)	<0·001
HbA1c (%; median)	4·6 (0·4)	4·6 (0·4)	0·386	4·7 (0·5)	4·7 (0·4)	<0·001
TG (mmol/L; median)	1·2 (0·8)	1·1 (0·7)	<0·001	1·2 (0·8)	1·1 (0·7)	<0·001
TC (mmol/L)	5·7 (1·0)	6·1 (1·1)	<0·001	5·5 (1·1)	6·0 (1·1)	<0·001
HDL-C (mmol/L)	1·3 (0·3)	1·6 (0·4)	<0·001	1·2 (0·3)	1·6 (0·4)	<0·001
LDL-C (mmol/L)	3·8 (0·9)	3·9 (1·0)	<0·001	3·7 (1·0)	3·9 (1·0)	<0·001
ApoA1 (g/L)	1·6 (0·3)	1·8 (0·3)	<0·001	1·5 (0·3)	1·7 (0·3)	<0·001
ApoB (g/L)	1·09 (0·24)	1·10 (0·24)	0·025	1·06 (0·24)	1·09 (0·24)	<0·001
CRP (mg/L; median)	1·6 (2·5)	1·6 (2·6)	0·890	1·6 (2·7)	1·7 (2·8)	0·690
Hb (g/L)	149·4 (10·2)	137·3 (9·6)	<0·001	148·6 (11·0)	137·0 (9·9)	<0·001
**Follow-up information (N [%] or Median [IQR])**						
Follow-up time (year)	9·2 (4·4)	9·6 (2·3)		13·0 (1·5)	13·0 (1·5)	
Number of incident cases	1852 (45·1%)	1829 (33·7%)		1455 (26·6%)	1219 (18·4%)	
Onset age of incident cases	71·9 (10·7)	72·7 (12·5)		81·1 (11·8)	82·5 (13·1)	

[1] Two twin pairs with unknown zygosity were not shown in the table.
[2] Individuals whose co-twins were not included
[3] Baseline survey refers to the blood sampling conducted in 2004-2008
[4] Including cancer, diabetes, MI, CHF, stroke, COPD, and dementia.

MZ monozygotic, DZ dizygotic, N number of individuals, SD standard deviation, MI myocardial infarction, CHF coronary heart failure, COPD chronic obstructive pulmonary disease, FBG fasting blood glucose, HbA1c hemoglobin A1C, TC total cholesterol, HDL-C high-density lipoprotein cholesterol, LDL-C low-density lipoprotein cholesterol, ApoA1 Apolipoprotein A1, ApoB Apolipoprotein B, TG triglyceride, CRP C-reactive protein, Hb hemoglobin.

Supplementary Table 5 Number of prevalent and incident events in all participants, men, and women

	Event	Number of individuals	Number of event-free individuals at baseline	Mean age at baseline	Median follow-up year	Number of incident cases	Proportion of incident cases	Median onset age of incident cases
All	Diabetes	12098	11616	64·8	9·8	831	7·2%	73·1
All	CHF	12098	11879	64·7	9·8	845	7·1%	78·9
All	MI	12098	11412	64·6	9·8	881	7·7%	74·5
All	Stroke	12098	11750	64·7	9·8	746	6·3%	76·7
All	Cancer	12098	10877	64·5	9·7	2303	21·2%	72·1
All	COPD	12098	11906	64·8	9·8	486	4·1%	73·8
All	Dementia	12098	12077	64·9	9·8	541	4·5%	80·4
All	Death up to 20161231	12098	12098	64·9	9·8	1788	14·8%	80·2
All	Any chronic disease	12098	9538	63·9	9·5	3681	38·6%	72·3
All	Death	12098	12098	64·9	13	2674	22·1%	81·7
Men	Diabetes	5469	5176	65·1	9·7	452	8·7%	72·8
Men	CHF	5469	5336	65·1	9·8	480	9%	78
Men	MI	5469	4967	64·8	9·7	564	11·4%	74·1
Men	Stroke	5469	5277	65·1	9·8	440	8·3%	76·2
Men	Cancer	5469	4933	64·8	9·6	1226	24·9%	72·5
Men	COPD	5469	5383	65·2	9·8	236	4·4%	74·6
Men	Dementia	5469	5454	65·2	9·8	247	4·5%	79·8
Men	Death up to 20161231	5469	5469	65·3	9·8	993	18·2%	80
Men	Any chronic disease	5469	4110	64	9·2	1852	45·1%	71·9
Men	Death	5469	5469	65·3	13	1455	26·6%	81·1
Women	Diabetes	6629	6440	64·5	9·8	379	5·9%	73·6
Women	CHF	6629	6543	64·5	9·8	365	5·6%	79·6
Women	MI	6629	6445	64·4	9·8	317	4·9%	75
Women	Stroke	6629	6473	64·5	9·8	306	4·7%	76·8
Women	Cancer	6629	5944	64·3	9·7	1077	18·1%	71·8
Women	COPD	6629	6523	64·5	9·8	250	3·8%	72·9

Women	Dementia	6629	6623	64·6	9·8	294	4·4%	80·7
Women	Death up to 20161231	6629	6629	64·6	9·8	795	12%	80·3
Women	Any chronic disease	6629	5428	63·8	9·6	1829	33·7%	72·7
Women	Death	6629	6629	64·6	13	1219	18·4%	82·5

MI myocardial infarction, CHF coronary heart failure, COPD chronic obstructive pulmonary disease.

Supplementary Table 6 Associations of serum clinical biomarkers with healthspan and lifespan in all participants, men, and women

Group	Outcome	Serum biomarker	Number of individuals	Number of events	Median follow-up year	HR (95%CI)	FDR-corrected P value	Raw P value
All	Any chronic disease	FBG	9538	3681	9·5	1·28 (1·23, 1·33)	5·15e-33	1·03e-33
All	Any chronic disease	HbA1c	9538	3681	9·5	1·29 (1·24, 1·34)	9·07e-37	9·07e-38
All	Any chronic disease	TG	9538	3681	9·5	1·07 (1·03, 1·11)	0·001	6·05e-04
All	Any chronic disease	TC	9538	3681	9·5	0·96 (0·92, 0·99)	0·022	0·015
All	Any chronic disease	LDL-C	9538	3681	9·5	0·96 (0·93, 1·00)	0·060	0·048
All	Any chronic disease	ApoB	9538	3681	9·5	1·00 (0·96, 1·03)	0·833	0·833
All	Any chronic disease	HDL-C	9538	3681	9·5	0·92 (0·89, 0·96)	1·51e-04	6·03e-05
All	Any chronic disease	ApoA1	9538	3681	9·5	0·93 (0·89, 0·96)	2·12e-04	1·06e-04
All	Any chronic disease	CRP	9538	3681	9·5	1·11 (1·08, 1·15)	2·21e-09	6·62e-10
All	Any chronic disease	Hb	9538	3681	9·5	0·99 (0·95, 1·03)	0·791	0·711
All	Death	FBG	12098	2674	13·0	1·18 (1·13, 1·22)	5·45e-17	1·09e-17
All	Death	HbA1c	12098	2674	13·0	1·22 (1·17, 1·26)	6·42e-28	6·42e-29
All	Death	TG	12098	2674	13·0	1·12 (1·07, 1·17)	1·76e-06	1·23e-06
All	Death	TC	12098	2674	13·0	0·90 (0·86, 0·94)	1·76e-06	1·07e-06
All	Death	LDL-C	12098	2674	13·0	0·89 (0·85, 0·93)	2·14e-07	8·56e-08
All	Death	ApoB	12098	2674	13·0	0·95 (0·91, 0·99)	0·016	0·016
All	Death	HDL-C	12098	2674	13·0	0·91 (0·86, 0·96)	2·74e-04	2·47e-04
All	Death	ApoA1	12098	2674	13·0	0·91 (0·87, 0·96)	1·72e-04	1·38e-04
All	Death	CRP	12098	2674	13·0	1·15 (1·10, 1·20)	8·17e-11	2·45e-11
All	Death	Hb	12098	2674	13·0	0·89 (0·85, 0·93)	6·40e-07	3·20e-07
Men	Any chronic disease	FBG	4110	1852	9·2	1·23 (1·16, 1·29)	4·50e-14	9·01e-15
Men	Any chronic disease	HbA1c	4110	1852	9·2	1·25 (1·19, 1·32)	1·77e-16	1·77e-17
Men	Any chronic disease	TG	4110	1852	9·2	1·08 (1·02, 1·14)	0·011	0·004

Men	Any chronic disease	TC	4110	1852	9·2	0·98 (0·93, 1·04)	0·599	0·534
Men	Any chronic disease	LDL-C	4110	1852	9·2	0·99 (0·94, 1·04)	0·599	0·599
Men	Any chronic disease	ApoB	4110	1852	9·2	1·02 (0·97, 1·08)	0·537	0·376
Men	Any chronic disease	HDL-C	4110	1852	9·2	0·92 (0·86, 0·98)	0·014	0·007
Men	Any chronic disease	ApoA1	4110	1852	9·2	0·93 (0·88, 0·98)	0·021	0·012
Men	Any chronic disease	CRP	4110	1852	9·2	1·09 (1·05, 1·15)	3·50e-04	1·05e-04
Men	Any chronic disease	Hb	4110	1852	9·2	0·99 (0·93, 1·04)	0·599	0·592
Women	Any chronic disease	FBG	5428	1829	9·6	1·35 (1·28, 1·43)	6·33e-24	6·33e-25
Women	Any chronic disease	HbA1c	5428	1829	9·6	1·35 (1·28, 1·44)	2·69e-23	5·38e-24
Women	Any chronic disease	TG	5428	1829	9·6	1·07 (1·01, 1·13)	0·038	0·027
Women	Any chronic disease	TC	5428	1829	9·6	0·93 (0·89, 0·98)	0·016	0·010
Women	Any chronic disease	LDL-C	5428	1829	9·6	0·95 (0·90, 1·00)	0·050	0·040
Women	Any chronic disease	ApoB	5428	1829	9·6	0·98 (0·93, 1·03)	0·380	0·342
Women	Any chronic disease	HDL-C	5428	1829	9·6	0·92 (0·88, 0·97)	0·006	0·002
Women	Any chronic disease	ApoA1	5428	1829	9·6	0·92 (0·88, 0·97)	0·006	0·003
Women	Any chronic disease	CRP	5428	1829	9·6	1·14 (1·08, 1·20)	8·04e-06	2·41e-06
Women	Any chronic disease	Hb	5428	1829	9·6	1·00 (0·95, 1·07)	0·892	0·892
Men	Death	FBG	5469	1455	13·0	1·18 (1·13, 1·24)	1·88e-11	3·76e-12
Men	Death	HbA1c	5469	1455	13·0	1·22 (1·17, 1·28)	1·47e-18	1·47e-19
Men	Death	TG	5469	1455	13·0	1·11 (1·05, 1·18)	4·31e-04	3·02e-04
Men	Death	TC	5469	1455	13·0	0·89 (0·84, 0·95)	3·52e-04	2·11e-04
Men	Death	LDL-C	5469	1455	13·0	0·88 (0·83, 0·94)	1·27e-04	5·06e-05
Men	Death	ApoB	5469	1455	13·0	0·96 (0·91, 1·02)	0·151	0·151
Men	Death	HDL-C	5469	1455	13·0	0·88 (0·82, 0·95)	0·002	0·002
Men	Death	ApoA1	5469	1455	13·0	0·90 (0·84, 0·96)	0·003	0·003
Men	Death	CRP	5469	1455	13·0	1·15 (1·09, 1·21)	5·83e-07	1·75e-07

Men	Death	Hb	5469	1455	13·0	0·89 (0·84, 0·95)	2·80e-04	1·40e-04
Women	Death	FBG	6629	1219	13·0	1·17 (1·10, 1·24)	2·77e-06	5·54e-07
Women	Death	HbA1c	6629	1219	13·0	1·20 (1·14, 1·27)	5·48e-10	5·48e-11
Women	Death	TG	6629	1219	13·0	1·11 (1·04, 1·19)	0·004	0·003
Women	Death	TC	6629	1219	13·0	0·90 (0·85, 0·96)	0·001	8·98e-04
Women	Death	LDL-C	6629	1219	13·0	0·90 (0·84, 0·95)	8·51e-04	4·25e-04
Women	Death	ApoB	6629	1219	13·0	0·94 (0·89, 1·00)	0·039	0·039
Women	Death	HDL-C	6629	1219	13·0	0·93 (0·87, 0·99)	0·033	0·029
Women	Death	ApoA1	6629	1219	13·0	0·92 (0·86, 0·98)	0·018	0·015
Women	Death	CRP	6629	1219	13·0	1·16 (1·09, 1·24)	1·78e-05	5·33e-06
Women	Death	Hb	6629	1219	13·0	0·88 (0·82, 0·94)	4·81e-04	1·93e-04

FBG fasting blood glucose, HbA1c hemoglobin A1C, TC total cholesterol, HDL-C high-density lipoprotein cholesterol, LDL-C low-density lipoprotein cholesterol, ApoA1 Apolipoprotein A1, ApoB Apolipoprotein B, TG triglyceride, CRP C-reactive protein, Hb hemoglobin.

Supplementary Table 7 Validation of biomarker-healthspan associations in SATSA

Biomarker	SATSA					Effect directions in SATSA and in TwinGene	HR (95%CI) in TwinGene
	Number of individuals	Number of chronic events	Median follow-up year	HR (95%CI)	Raw P value		
Serum							
HbA1c	117	34	3.3	1·13 (0·56, 2·27)	0·727	- -	1·29 (1·24, 1·34)
FBG	759	642	14.9	1·18 (1·09, 1·29)	1·36e-4	- - *	1·28 (1·23, 1·33)
CRP	242	142	7.7	1·21 (1·03, 1·41)	0·018	- - *	1·11 (1·08, 1·15)
TG	735	619	15.0	1·06 (0·97, 1·16)	0·176	- -	1·07 (1·03, 1·11)
TC	735	619	15.0	1·03 (0·96, 1·11)	0·421	+ -	0·96 (0·92, 0·99)
ApoA1	737	621	14.9	0·94 (0·86, 1·02)	0·153	+ +	0·93 (0·89, 0·96)
HDL-C	687	576	14.0	0·89 (0·82, 0·96)	0·002	+ + *	0·92 (0·89, 0·96)
PRS							
FBG	492	406	15.3	1·04 (0·95, 1·15)	0·382	- -	1·05 (1·02, 1·09)

HRs indicate the relative risk of experiencing any chronic event associated with one-SD increase in serum biomarker concentrations or FBG PRS.
Significant effects (- risk factor, + protective factor) found in both TwinGene (FDR-adjusted P<0.05) and SATSA (raw P<0.05) were marked by *.
HbA1c hemoglobin A1C, FBG fasting blood glucose, CRP C-reactive protein, TG triglyceride, TC total cholesterol, ApoA1 Apolipoprotein A1, HDL-C high-density lipoprotein cholesterol, PRS polygenic risk score, HR hazard ratio, CI confidence interval.

Supplementary Table 8 Multiple-biomarkers regression

Serum biomarkers included in the final model	HR (95%CI)	Raw P value
Healthspan model		
FBG	1·14 (1·08, 1·20)	1·54e-06
HbA1c	1·17 (1·11, 1·23)	5·95e-09
TG	1·05 (1·01, 1·09)	0·020
TC	0·86 (0·77, 0·96)	0·008
LDL-C	1·11 (1·00, 1·24)	0·051
CRP	1·10 (1·07, 1·14)	1·65e-08
Lifespan model		
FBG	1·04 (0·99, 1·10)	0·121
HbA1c	1·14 (1·08, 1·20)	6·23e-07
TG	1·12 (1·07, 1·17)	3·42e-06
TC	0·91 (0·88, 0·95)	1·87e-05
Hb	0·92 (0·88, 0·96)	1·08e-04
CRP	1·12 (1·08, 1·17)	2·15e-08

HRs indicate the relative risk of experiencing any chronic event associated with one-SD increase in serum biomarker concentrations or FBG PRS.
HbA1c hemoglobin A1C, FBG fasting blood glucose, CRP C-reactive protein, TG triglyceride, TC total cholesterol, Hb hemoglobin, LDL-C low-density lipoprotein cholesterol, HR hazard ratio, CI confidence interval.

Supplementary Table 9 Associations of biomarker PRSs with healthspan and lifespan in all participants, men, and women

Group	Outcome	PRS	Number of individuals	Number of events	Median follow-up year	HR (95%CI)	FDR-corrected P value	Raw P value
All	Any chronic disease	FBG	7972	3104	9·3	1·05 (1·02, 1·09)	0·050	0·005
All	Any chronic disease	HbA1c	7972	3104	9·3	1·04 (1·00, 1·08)	0·255	0·051
All	Any chronic disease	TG	7972	3104	9·3	1·01 (0·98, 1·05)	0·706	0·423
All	Any chronic disease	TC	7972	3104	9·3	1·02 (0·99, 1·06)	0·630	0·221
All	Any chronic disease	LDL-C	7972	3104	9·3	1·02 (0·99, 1·06)	0·630	0·252
All	Any chronic disease	ApoB	7972	3104	9·3	1·02 (0·98, 1·05)	0·706	0·416
All	Any chronic disease	HDL-C	7972	3104	9·3	1·00 (0·96, 1·03)	0·948	0·948
All	Any chronic disease	ApoA1	7972	3104	9·3	1·00 (0·96, 1·03)	0·922	0·829
All	Any chronic disease	CRP	7972	3104	9·3	0·99 (0·96, 1·03)	0·922	0·732
All	Any chronic disease	Hb	7972	3104	9·3	0·99 (0·96, 1·03)	0·922	0·780
Men	Any chronic disease	FBG	3572	1649	9·2	1·03 (0·98, 1·08)	0·497	0·212
Men	Any chronic disease	HbA1c	3572	1649	9·2	1·02 (0·97, 1·07)	0·850	0·532
Men	Any chronic disease	TG	3572	1649	9·2	1·03 (0·98, 1·08)	0·497	0·191
Men	Any chronic disease	TC	3572	1649	9·2	1·03 (0·98, 1·09)	0·497	0·248
Men	Any chronic disease	LDL-C	3572	1649	9·2	1·03 (0·98, 1·09)	0·497	0·207
Men	Any chronic disease	ApoB	3572	1649	9·2	1·04 (0·99, 1·09)	0·497	0·159
Men	Any chronic disease	HDL-C	3572	1649	9·2	0·99 (0·95, 1·04)	0·946	0·799
Men	Any chronic disease	ApoA1	3572	1649	9·2	1·00 (0·96, 1·05)	0·946	0·942
Men	Any chronic disease	CRP	3572	1649	9·2	1·00 (0·95, 1·05)	0·946	0·946
Men	Any chronic disease	Hb	3572	1649	9·2	0·99 (0·94, 1·04)	0·850	0·595
Women	Any chronic disease	FBG	4400	1455	9·6	1·08 (1·02, 1·14)	0·046	0·005
Women	Any chronic disease	HbA1c	4400	1455	9·6	1·06 (1·01, 1·12)	0·123	0·025
Women	Any chronic disease	TG	4400	1455	9·6	0·99 (0·94, 1·05)	0·871	0·828

Women	Any chronic disease	TC	4400	1455	9·6	1·02 (0·97, 1·07)	0·871	0·504
Women	Any chronic disease	LDL-C	4400	1455	9·6	1·01 (0·96, 1·06)	0·871	0·690
Women	Any chronic disease	ApoB	4400	1455	9·6	1·00 (0·95, 1·05)	0·871	0·871
Women	Any chronic disease	HDL-C	4400	1455	9·6	1·01 (0·96, 1·06)	0·871	0·793
Women	Any chronic disease	ApoA1	4400	1455	9·6	0·99 (0·94, 1·04)	0·871	0·726
Women	Any chronic disease	CRP	4400	1455	9·6	0·99 (0·94, 1·04)	0·871	0·683
Women	Any chronic disease	Hb	4400	1455	9·6	1·01 (0·96, 1·06)	0·871	0·772
All	Death	FBG	10098	2264	13·0	1·00 (0·96, 1·05)	0·867	0·867
All	Death	HbA1c	10098	2264	13·0	1·01 (0·96, 1·05)	0·867	0·805
All	Death	TG	10098	2264	13·0	1·03 (0·99, 1·08)	0·251	0·126
All	Death	TC	10098	2264	13·0	1·05 (1·01, 1·10)	0·064	0·019
All	Death	LDL-C	10098	2264	13·0	1·05 (1·01, 1·10)	0·064	0·017
All	Death	ApoB	10098	2264	13·0	1·05 (1·01, 1·09)	0·066	0·026
All	Death	HDL-C	10098	2264	13·0	0·99 (0·94, 1·03)	0·643	0·514
All	Death	ApoA1	10098	2264	13·0	0·98 (0·94, 1·03)	0·643	0·452
All	Death	CRP	10098	2264	13·0	0·91 (0·87, 0·95)	1·54e-04	1·54e-05
All	Death	Hb	10098	2264	13·0	0·98 (0·94, 1·02)	0·643	0·416
Men	Death	FBG	4775	1309	13·0	1·00 (0·95, 1·06)	0·966	0·966
Men	Death	HbA1c	4775	1309	13·0	1·03 (0·98, 1·09)	0·330	0·264
Men	Death	TG	4775	1309	13·0	1·06 (1·00, 1·11)	0·240	0·048
Men	Death	TC	4775	1309	13·0	1·04 (0·98, 1·10)	0·329	0·200
Men	Death	LDL-C	4775	1309	13·0	1·05 (0·99, 1·11)	0·255	0·099
Men	Death	ApoB	4775	1309	13·0	1·05 (0·99, 1·11)	0·255	0·102
Men	Death	HDL-C	4775	1309	13·0	0·97 (0·91, 1·02)	0·329	0·229
Men	Death	ApoA1	4775	1309	13·0	0·97 (0·91, 1·02)	0·329	0·231
Men	Death	CRP	4775	1309	13·0	0·92 (0·86, 0·97)	0·048	0·005

Men	Death	Hb	4775	1309	13·0	0·99 (0·93, 1·04)	0·697	0·627
Women	Death	FBG	5323	955	13·0	1·01 (0·95, 1·08)	0·801	0·656
Women	Death	HbA1c	5323	955	13·0	0·97 (0·91, 1·04)	0·801	0·411
Women	Death	TG	5323	955	13·0	1·00 (0·94, 1·07)	0·984	0·984
Women	Death	TC	5323	955	13·0	1·07 (1·01, 1·14)	0·148	0·030
Women	Death	LDL-C	5323	955	13·0	1·06 (1·00, 1·13)	0·212	0·064
Women	Death	ApoB	5323	955	13·0	1·05 (0·99, 1·12)	0·249	0·099
Women	Death	HDL-C	5323	955	13·0	1·02 (0·95, 1·09)	0·801	0·576
Women	Death	ApoA1	5323	955	13·0	1·01 (0·95, 1·08)	0·801	0·721
Women	Death	CRP	5323	955	13·0	0·89 (0·84, 0·95)	0·008	7·79e-04
Women	Death	Hb	5323	955	13·0	0·98 (0·92, 1·05)	0·801	0·579

FBG fasting blood glucose, HbA1c hemoglobin A1C, TC total cholesterol, HDL-C high-density lipoprotein cholesterol, LDL-C low-density lipoprotein cholesterol, ApoA1 Apolipoprotein A1, ApoB Apolipoprotein B, TG triglyceride, CRP C-reactive protein, Hb hemoglobin.

Supplementary Table 10 Associations of regional CRP PRS and death

	HR (95% CI)	P values
CRP PRS and death		
Genome-wide PRS derived from 95 SNPs	0·91 (0·87, 0·95)	1·54e-05
Regional CRP PRS derived from 4 SNPs [1]	0·94 (0·90, 0·98)	0·004

[1] Derived from rs3093077, rs1205, rs1130864, rs1800947 (*CRP*).

Supplementary Table 11 Leave-one-disease-out sensitivity analysis

Group	Biomarker	Number of individuals	Number of events	Median follow-up years	HR (95%CI)	FDR-adjusted P value	Raw P value
Serum Biomarker							
All events	FBG	9538	3681	9·5	1·28 (1·23, 1·33)	5·15e-33	1·03e-33
All events	HbA1c	9538	3681	9·5	1·29 (1·24, 1·34)	9·07e-37	9·07e-38
All events	TG	9538	3681	9·5	1·07 (1·03, 1·11)	0·001	6·05e-04
All events	TC	9538	3681	9·5	0·96 (0·92, 0·99)	0·022	0·015
All events	LDL-C	9538	3681	9·5	0·96 (0·93, 1·00)	0·060	0·048
All events	ApoB	9538	3681	9·5	1·00 (0·96, 1·03)	0·833	0·833
All events	HDL-C	9538	3681	9·5	0·92 (0·89, 0·96)	1·51e-04	6·03e-05
All events	ApoA1	9538	3681	9·5	0·93 (0·89, 0·96)	2·12e-04	1·06e-04
All events	CRP	9538	3681	9·5	1·11 (1·08, 1·15)	2·21e-09	6·62e-10
All events	Hb	9538	3681	9·5	0·99 (0·95, 1·03)	0·791	0·711
Exclude cancer	FBG	9538	2591	9·7	1·44 (1·38, 1·51)	6·94e-55	1·39e-55
Exclude cancer	HbA1c	9538	2591	9·7	1·44 (1·38, 1·51)	1·04e-56	1·04e-57
Exclude cancer	TG	9538	2591	9·7	1·14 (1·09, 1·19)	1·68e-08	6·72e-09
Exclude cancer	TC	9538	2591	9·7	0·94 (0·90, 0·98)	0·005	0·004
Exclude cancer	LDL-C	9538	2591	9·7	0·94 (0·90, 0·98)	0·009	0·007
Exclude cancer	ApoB	9538	2591	9·7	1·01 (0·96, 1·05)	0·801	0·801
Exclude cancer	HDL-C	9538	2591	9·7	0·87 (0·83, 0·92)	1·27e-07	6·37e-08
Exclude cancer	ApoA1	9538	2591	9·7	0·89 (0·85, 0·94)	4·80e-06	2·88e-06
Exclude cancer	CRP	9538	2591	9·7	1·14 (1·10, 1·19)	2·73e-10	8·18e-11
Exclude cancer	Hb	9538	2591	9·7	1·02 (0·97, 1·07)	0·465	0·419
Exclude diabetes	FBG	9538	3419	9·6	1·05 (1·00, 1·09)	0·065	0·033
Exclude diabetes	HbA1c	9538	3419	9·6	1·06 (1·01, 1·10)	0·029	0·009
Exclude diabetes	TG	9538	3419	9·6	1·04 (1·00, 1·08)	0·079	0·048

Exclude diabetes	TC	9538	3419	9·6	0·99 (0·96, 1·03)	0·827	0·744
Exclude diabetes	LDL-C	9538	3419	9·6	1·00 (0·97, 1·04)	0·923	0·923
Exclude diabetes	ApoB	9538	3419	9·6	1·02 (0·98, 1·05)	0·540	0·378
Exclude diabetes	HDL-C	9538	3419	9·6	0·95 (0·92, 0·99)	0·049	0·020
Exclude diabetes	ApoA1	9538	3419	9·6	0·95 (0·91, 0·99)	0·029	0·007
Exclude diabetes	CRP	9538	3419	9·6	1·11 (1·07, 1·15)	1·17e-07	1·17e-08
Exclude diabetes	Hb	9538	3419	9·6	0·99 (0·95, 1·03)	0·653	0·522
Exclude MI	FBG	9538	3456	9·6	1·32 (1·27, 1·38)	3·55e-41	7·09e-42
Exclude MI	HbA1c	9538	3456	9·6	1·33 (1·27, 1·38)	4·70e-42	4·70e-43
Exclude MI	TG	9538	3456	9·6	1·07 (1·03, 1·11)	0·002	0·001
Exclude MI	TC	9538	3456	9·6	0·94 (0·90, 0·98)	0·002	0·001
Exclude MI	LDL-C	9538	3456	9·6	0·94 (0·91, 0·98)	0·002	0·002
Exclude MI	ApoB	9538	3456	9·6	0·98 (0·94, 1·01)	0·200	0·180
Exclude MI	HDL-C	9538	3456	9·6	0·93 (0·90, 0·97)	0·002	9·64e-04
Exclude MI	ApoA1	9538	3456	9·6	0·93 (0·90, 0·97)	0·001	5·52e-04
Exclude MI	CRP	9538	3456	9·6	1·11 (1·08, 1·15)	9·31e-09	2·79e-09
Exclude MI	Hb	9538	3456	9·6	0·99 (0·95, 1·03)	0·594	0·594
Exclude stroke	FBG	9538	3506	9·6	1·31 (1·26, 1·37)	1·14e-39	2·28e-40
Exclude stroke	HbA1c	9538	3506	9·6	1·33 (1·27, 1·38)	1·07e-42	1·07e-43
Exclude stroke	TG	9538	3506	9·6	1·08 (1·04, 1·12)	3·07e-04	1·84e-04
Exclude stroke	TC	9538	3506	9·6	0·96 (0·92, 0·99)	0·026	0·018
Exclude stroke	LDL-C	9538	3506	9·6	0·96 (0·93, 1·00)	0·058	0·046
Exclude stroke	ApoB	9538	3506	9·6	1·00 (0·96, 1·04)	0·966	0·966
Exclude stroke	HDL-C	9538	3506	9·6	0·92 (0·88, 0·96)	8·36e-05	4·05e-05
Exclude stroke	ApoA1	9538	3506	9·6	0·92 (0·88, 0·96)	8·36e-05	4·18e-05
Exclude stroke	CRP	9538	3506	9·6	1·11 (1·08, 1·16)	6·33e-09	1·90e-09

Exclude stroke	Hb	9538	3506	9·6	1·00 (0·96, 1·04)	0·966	0·911
Exclude CHF	FBG	9538	3578	9·6	1·29 (1·24, 1·35)	1·28e-35	2·56e-36
Exclude CHF	HbA1c	9538	3578	9·6	1·31 (1·26, 1·36)	2·34e-39	2·34e-40
Exclude CHF	TG	9538	3578	9·6	1·08 (1·04, 1·12)	2·63e-04	1·58e-04
Exclude CHF	TC	9538	3578	9·6	0·96 (0·92, 0·99)	0·022	0·016
Exclude CHF	LDL-C	9538	3578	9·6	0·96 (0·93, 1·00)	0·068	0·055
Exclude CHF	ApoB	9538	3578	9·6	1·00 (0·96, 1·03)	0·927	0·927
Exclude CHF	HDL-C	9538	3578	9·6	0·91 (0·88, 0·95)	3·12e-05	1·25e-05
Exclude CHF	ApoA1	9538	3578	9·6	0·92 (0·89, 0·96)	1·09e-04	5·43e-05
Exclude CHF	CRP	9538	3578	9·6	1·11 (1·07, 1·15)	1·27e-08	3·80e-09
Exclude CHF	Hb	9538	3578	9·6	1·00 (0·96, 1·04)	0·927	0·870
Exclude COPD	FBG	9538	3585	9·6	1·28 (1·23, 1·33)	7·43e-33	1·49e-33
Exclude COPD	HbA1c	9538	3585	9·6	1·30 (1·25, 1·35)	1·89e-37	1·89e-38
Exclude COPD	TG	9538	3585	9·6	1·06 (1·02, 1·10)	0·003	0·002
Exclude COPD	TC	9538	3585	9·6	0·96 (0·92, 0·99)	0·030	0·021
Exclude COPD	LDL-C	9538	3585	9·6	0·97 (0·93, 1·00)	0·108	0·086
Exclude COPD	ApoB	9538	3585	9·6	1·00 (0·96, 1·03)	0·876	0·876
Exclude COPD	HDL-C	9538	3585	9·6	0·92 (0·88, 0·96)	1·07e-04	4·27e-05
Exclude COPD	ApoA1	9538	3585	9·6	0·92 (0·89, 0·96)	1·42e-04	7·12e-05
Exclude COPD	CRP	9538	3585	9·6	1·11 (1·07, 1·15)	2·10e-08	6·31e-09
Exclude COPD	Hb	9538	3585	9·6	0·98 (0·94, 1·02)	0·468	0·421
Exclude dementia	FBG	9538	3574	9·6	1·29 (1·24, 1·34)	1·54e-34	3·09e-35
Exclude dementia	HbA1c	9538	3574	9·6	1·31 (1·26, 1·36)	2·53e-39	2·53e-40
Exclude dementia	TG	9538	3574	9·6	1·07 (1·03, 1·11)	0·001	7·33e-04
Exclude dementia	TC	9538	3574	9·6	0·95 (0·91, 0·98)	0·006	0·004
Exclude dementia	LDL-C	9538	3574	9·6	0·96 (0·92, 0·99)	0·020	0·016

Exclude dementia	ApoB	9538	3574	9·6	0·99 (0·96, 1·03)	0·644	0·579
Exclude dementia	HDL-C	9538	3574	9·6	0·92 (0·88, 0·96)	8·08e-05	3·23e-05
Exclude dementia	ApoA1	9538	3574	9·6	0·93 (0·89, 0·96)	2·90e-04	1·45e-04
Exclude dementia	CRP	9538	3574	9·6	1·12 (1·09, 1·16)	1·53e-10	4·60e-11
Exclude dementia	Hb	9538	3574	9·6	1·00 (0·96, 1·04)	0·947	0·947
Biomarker PRS							
All events	FBG	7972	3104	9·3	1·05 (1·02, 1·09)	0·050	0·005
All events	HbA1c	7972	3104	9·3	1·04 (1·00, 1·08)	0·255	0·051
All events	TG	7972	3104	9·3	1·01 (0·98, 1·05)	0·706	0·423
All events	TC	7972	3104	9·3	1·02 (0·99, 1·06)	0·630	0·221
All events	LDL-C	7972	3104	9·3	1·02 (0·99, 1·06)	0·630	0·252
All events	ApoB	7972	3104	9·3	1·02 (0·98, 1·05)	0·706	0·416
All events	HDL-C	7972	3104	9·3	1·00 (0·96, 1·03)	0·948	0·948
All events	ApoA1	7972	3104	9·3	1·00 (0·96, 1·03)	0·922	0·829
All events	CRP	7972	3104	9·3	0·99 (0·96, 1·03)	0·922	0·732
All events	Hb	7972	3104	9·3	0·99 (0·96, 1·03)	0·922	0·780
Exclude cancer	FBG	7972	2201	9·7	1·08 (1·04, 1·13)	0·002	1·84e-04
Exclude cancer	HbA1c	7972	2201	9·7	1·04 (0·99, 1·08)	0·175	0·087
Exclude cancer	TG	7972	2201	9·7	1·03 (0·99, 1·07)	0·273	0·191
Exclude cancer	TC	7972	2201	9·7	1·07 (1·02, 1·11)	0·024	0·005
Exclude cancer	LDL-C	7972	2201	9·7	1·06 (1·01, 1·10)	0·045	0·014
Exclude cancer	ApoB	7972	2201	9·7	1·05 (1·01, 1·10)	0·062	0·025
Exclude cancer	HDL-C	7972	2201	9·7	0·99 (0·95, 1·04)	0·859	0·784
Exclude cancer	ApoA1	7972	2201	9·7	0·99 (0·95, 1·04)	0·859	0·781
Exclude cancer	CRP	7972	2201	9·7	0·97 (0·92, 1·01)	0·185	0·111
Exclude cancer	Hb	7972	2201	9·7	1·00 (0·96, 1·05)	0·859	0·859

Exclude diabetes	FBG	7972	2874	9·6	1·02 (0·99, 1·06)	0·882	0·220
Exclude diabetes	HbA1c	7972	2874	9·6	1·01 (0·97, 1·05)	0·882	0·717
Exclude diabetes	TG	7972	2874	9·6	1·02 (0·98, 1·05)	0·882	0·420
Exclude diabetes	TC	7972	2874	9·6	1·01 (0·97, 1·05)	0·882	0·552
Exclude diabetes	LDL-C	7972	2874	9·6	1·01 (0·98, 1·05)	0·882	0·474
Exclude diabetes	ApoB	7972	2874	9·6	1·00 (0·97, 1·04)	0·882	0·793
Exclude diabetes	HDL-C	7972	2874	9·6	1·00 (0·97, 1·04)	0·946	0·946
Exclude diabetes	ApoA1	7972	2874	9·6	0·99 (0·96, 1·03)	0·882	0·786
Exclude diabetes	CRP	7972	2874	9·6	0·99 (0·95, 1·03)	0·882	0·646
Exclude diabetes	Hb	7972	2874	9·6	0·99 (0·95, 1·03)	0·882	0·586
Exclude MI	FBG	7972	2913	9·5	1·05 (1·01, 1·09)	0·092	0·009
Exclude MI	HbA1c	7972	2913	9·5	1·04 (1·00, 1·08)	0·233	0·047
Exclude MI	TG	7972	2913	9·5	1·01 (0·98, 1·05)	0·950	0·531
Exclude MI	TC	7972	2913	9·5	1·01 (0·97, 1·05)	0·950	0·549
Exclude MI	LDL-C	7972	2913	9·5	1·01 (0·97, 1·05)	0·950	0·665
Exclude MI	ApoB	7972	2913	9·5	1·00 (0·97, 1·04)	0·993	0·893
Exclude MI	HDL-C	7972	2913	9·5	1·00 (0·96, 1·04)	0·993	0·993
Exclude MI	ApoA1	7972	2913	9·5	1·00 (0·96, 1·04)	0·993	0·961
Exclude MI	CRP	7972	2913	9·5	0·99 (0·95, 1·03)	0·950	0·635
Exclude MI	Hb	7972	2913	9·5	0·98 (0·95, 1·02)	0·950	0·413
Exclude stroke	FBG	7972	2955	9·5	1·05 (1·01, 1·09)	0·147	0·015
Exclude stroke	HbA1c	7972	2955	9·5	1·04 (1·00, 1·08)	0·277	0·055
Exclude stroke	TG	7972	2955	9·5	1·01 (0·97, 1·04)	0·854	0·769
Exclude stroke	TC	7972	2955	9·5	1·02 (0·98, 1·05)	0·726	0·406
Exclude stroke	LDL-C	7972	2955	9·5	1·02 (0·98, 1·06)	0·726	0·284
Exclude stroke	ApoB	7972	2955	9·5	1·01 (0·98, 1·05)	0·726	0·491

Exclude stroke	HDL-C	7972	2955	9·5	0·99 (0·96, 1·03)	0·829	0·663
Exclude stroke	ApoA1	7972	2955	9·5	0·99 (0·95, 1·02)	0·726	0·438
Exclude stroke	CRP	7972	2955	9·5	0·99 (0·95, 1·02)	0·726	0·508
Exclude stroke	Hb	7972	2955	9·5	1·00 (0·96, 1·03)	0·866	0·866
Exclude CHF	FBG	7972	3025	9·5	1·05 (1·02, 1·09)	0·043	0·004
Exclude CHF	HbA1c	7972	3025	9·5	1·04 (1·00, 1·08)	0·160	0·032
Exclude CHF	TG	7972	3025	9·5	1·01 (0·98, 1·05)	0·704	0·466
Exclude CHF	TC	7972	3025	9·5	1·02 (0·98, 1·06)	0·704	0·311
Exclude CHF	LDL-C	7972	3025	9·5	1·02 (0·98, 1·05)	0·704	0·424
Exclude CHF	ApoB	7972	3025	9·5	1·01 (0·98, 1·05)	0·704	0·525
Exclude CHF	HDL-C	7972	3025	9·5	1·00 (0·96, 1·04)	0·978	0·978
Exclude CHF	ApoA1	7972	3025	9·5	1·00 (0·96, 1·03)	0·978	0·915
Exclude CHF	CRP	7972	3025	9·5	0·99 (0·95, 1·03)	0·704	0·563
Exclude CHF	Hb	7972	3025	9·5	0·99 (0·95, 1·02)	0·704	0·515
Exclude COPD	FBG	7972	3025	9·5	1·06 (1·02, 1·09)	0·039	0·004
Exclude COPD	HbA1c	7972	3025	9·5	1·05 (1·01, 1·09)	0·069	0·014
Exclude COPD	TG	7972	3025	9·5	1·01 (0·98, 1·05)	0·875	0·525
Exclude COPD	TC	7972	3025	9·5	1·03 (0·99, 1·07)	0·352	0·116
Exclude COPD	LDL-C	7972	3025	9·5	1·03 (0·99, 1·07)	0·352	0·141
Exclude COPD	ApoB	7972	3025	9·5	1·02 (0·98, 1·06)	0·598	0·299
Exclude COPD	HDL-C	7972	3025	9·5	1·00 (0·97, 1·04)	0·943	0·862
Exclude COPD	ApoA1	7972	3025	9·5	1·00 (0·96, 1·04)	0·943	0·943
Exclude COPD	CRP	7972	3025	9·5	1·00 (0·96, 1·03)	0·943	0·879
Exclude COPD	Hb	7972	3025	9·5	0·99 (0·96, 1·03)	0·943	0·754
Exclude dementia	FBG	7972	3016	9·5	1·05 (1·01, 1·09)	0·061	0·006
Exclude dementia	HbA1c	7972	3016	9·5	1·04 (1·00, 1·08)	0·138	0·028

Exclude dementia	TG	7972	3016	9·5	1·01 (0·98, 1·05)	0·943	0·516
Exclude dementia	TC	7972	3016	9·5	1·02 (0·98, 1·06)	0·943	0·350
Exclude dementia	LDL-C	7972	3016	9·5	1·02 (0·98, 1·05)	0·943	0·378
Exclude dementia	ApoB	7972	3016	9·5	1·01 (0·97, 1·05)	0·943	0·572
Exclude dementia	HDL-C	7972	3016	9·5	1·00 (0·97, 1·04)	0·996	0·996
Exclude dementia	ApoA1	7972	3016	9·5	1·00 (0·97, 1·04)	0·996	0·982
Exclude dementia	CRP	7972	3016	9·5	1·01 (0·97, 1·04)	0·943	0·755
Exclude dementia	Hb	7972	3016	9·5	0·99 (0·96, 1·03)	0·943	0·713

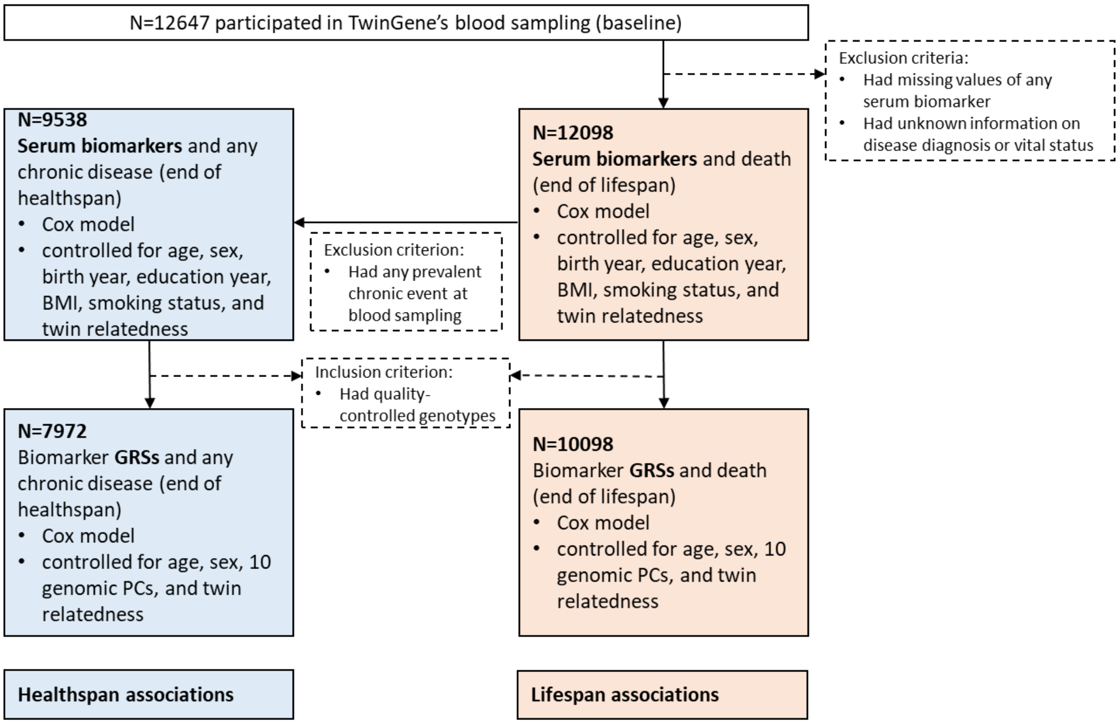

Supplementary Figure 1 Study design and study population

Of all TwinGene participants, a total of 12098 and 2024 qualified individuals had serum clinical biomarkers and serum non-targeted metabolites assessed, respectively. Individuals who had a previous diagnosis of any event were excluded in the healthspan analyses. Of all included participants, 10098 of them had quality controlled genotype data and were included in the genetic association analyses.

BMI body mass index, PRS polygenic risk score, PC principal component

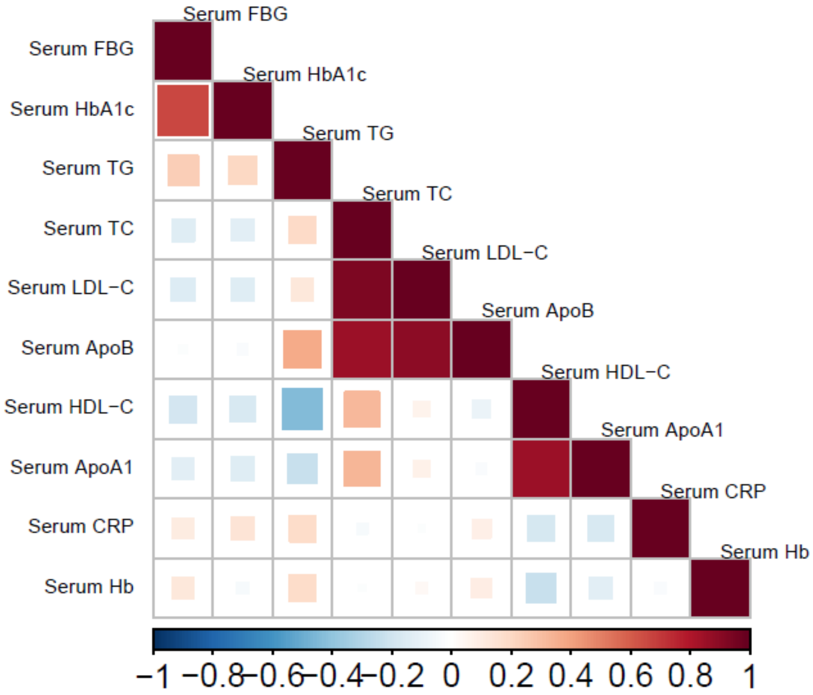

Supplementary Figure 2 Correlations of serum biomarkers

Cells of the heatmap represent the correlations between serum biomarker pairs, Color density of each cell indicates the magnitude of Pearson correlation coefficients, with red and blue showing positive and negative correlations, respectively. Glycemic and lipid biomarkers constituted three clusters of high correlations, namely 1) FBG and HbA1c, 2) TC, LDL-C, and ApoB, and 3) HDL-C and ApoA1·

FBG fasting blood glucose, HbA1c hemoglobin A1C, TC total cholesterol, HDL-C high-density lipoprotein cholesterol, LDL-C low-density lipoprotein cholesterol, ApoA1 Apolipoprotein A1, ApoB Apolipoprotein B, TG triglyceride, CRP C-reactive protein, Hb hemoglobin.

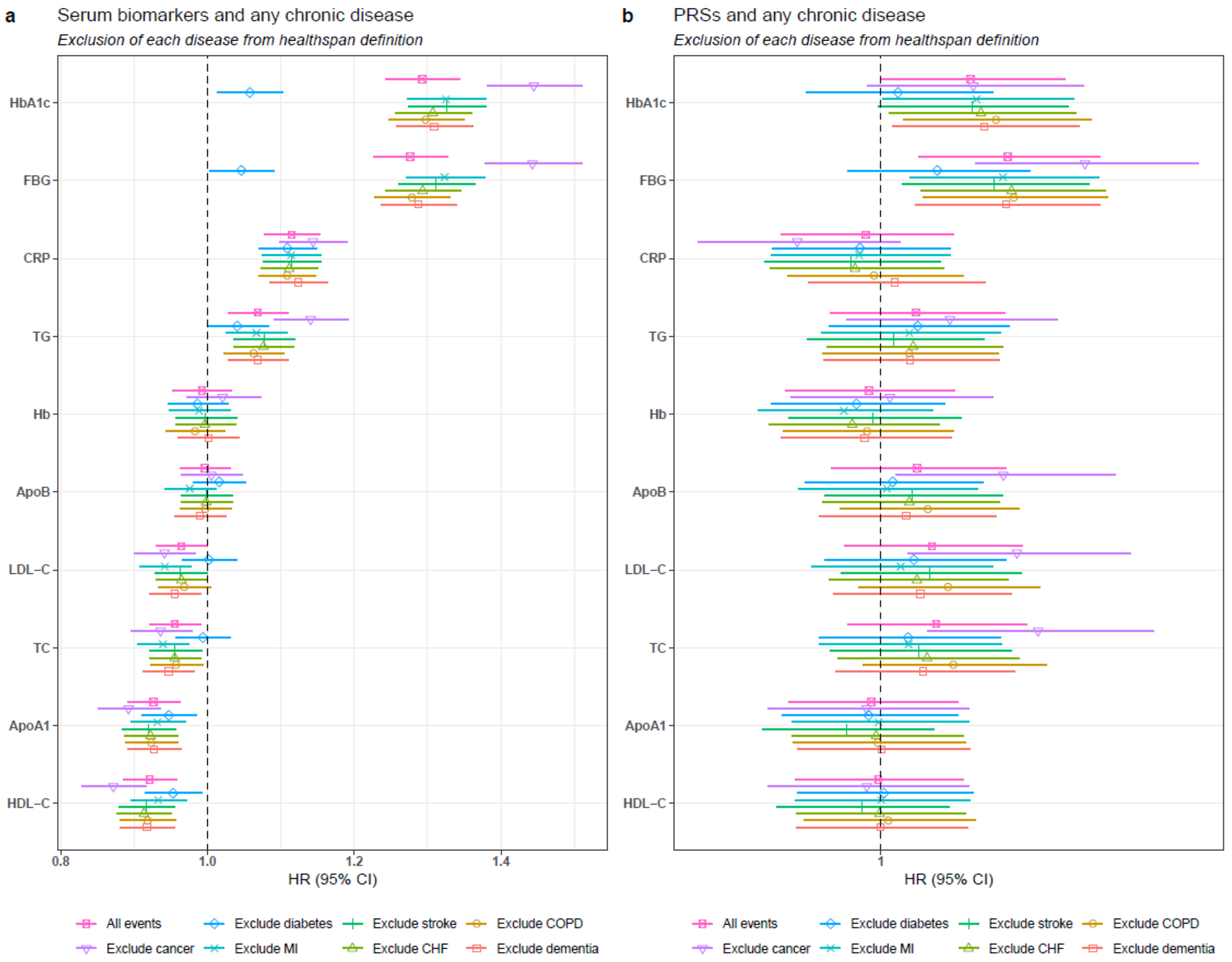

Supplementary Figure 3 Leave-one-disease-out sensitivity analysis

Supplementary Figure 3 shows the HR (95% CI) of any chronic disease that are associated with one-SD increase in serum biomarker concentration (Panel a) and PRS (Panel b). For each relationship, we estimated the relative risks among all participants and among subsets of study population after exclusion of individuals whose healthspan were ended due to the same disease.

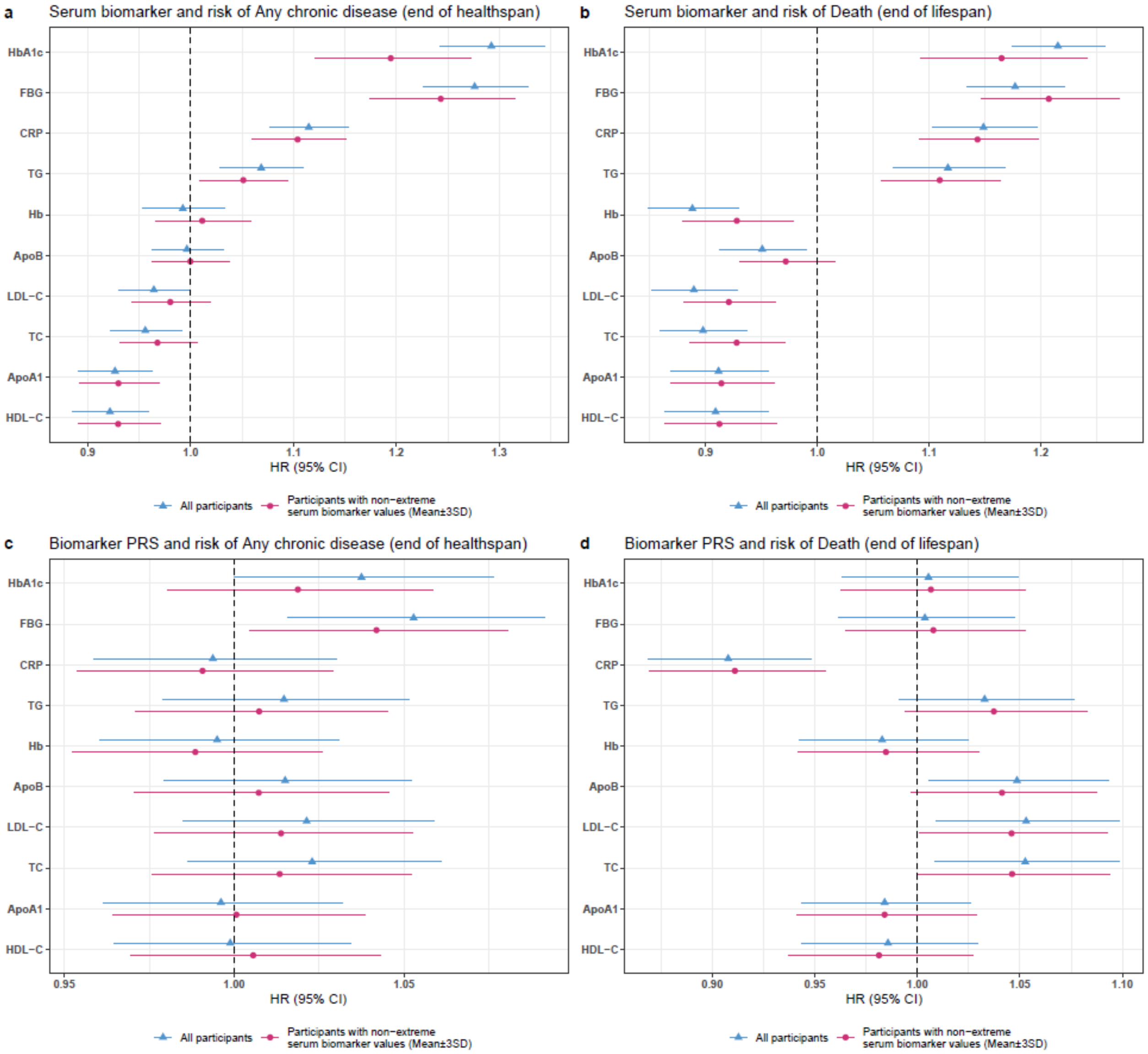

Supplementary Figure 4 Sensitivity analysis with the exclusion of serum biomarker outliers

Panel a-d illustrated the associations, quantified as HR (95% CI), of biomarkers with any chronic disease and death, respectively. HRs indicate the relative risk of experiencing any chronic disease or death associated with one-SD increase in serum biomarker concentrations or biomarker PRSs. Results estimated among the whole study population and among the non-outlier participants were showed in color blue and red, respectively.

Reference

1. Finkel D, Pedersen NL. Processing speed and longitudinal trajectories of change for cognitive abilities: The Swedish Adoption/Twin Study of Aging. *Aging Neuropsychol C* 2004; **11**(2-3): 325-45.
