# Supplementary material for: Clinical biomarkers and associations with healthspan and lifespan: Evidence from observational and genetic data
Source: eBioMedicine. 2021 Apr 1;66:103318. doi: 10.1016/j.ebiom.2021.103318 (PMC8047464; doi:10.1016/j.ebiom.2021.103318)
Supplement: Supplementary file 2 [file mmc2.docx]

**Supplementary files**

Supplementary Method 1 Validation in SATSA

Supplementary Table 1 ICD codes related to the chronic diseases

Supplementary Table 2 Phenotype codes and data linkage of UKB summary statistics

Supplementary Table 3 Serum biomarkers and PRSs

Supplementary Table 4 Sex-specific characteristics of study participants

Supplementary Table 5 Number of prevalent and incident events in all participants, men, and women

Supplementary Table 6 Associations of serum clinical biomarkers with healthspan and lifespan in all participants, men, and women

Supplementary Table 7 Validation of biomarker-healthspan associations in SATSA

Supplementary Table 8 Multiple-biomarkers regression

Supplementary Table 9 Associations of biomarker PRSs with healthspan and lifespan in all participants, men, and women

Supplementary Table 10 Associations of regional CRP PRS and death

Supplementary Table 11 Leave-one-disease-out sensitivity analysis

Supplementary Figure 1 Study design and study population

Supplementary Figure 2 Correlations of serum biomarkers

Supplementary Figure 3 Leave-one-disease-out sensitivity analysis

Supplementary Figure 4 Sensitivity analysis with the exclusion of serum biomarker outliers
